# DNGR-1-tracing marks an ependymal cell subset with damage-responsive neural stem cell potential

**DOI:** 10.1016/j.devcel.2022.07.012

**Published:** 2022-08-22

**Authors:** Bruno Frederico, Isaura Martins, Diana Chapela, Francesca Gasparrini, Probir Chakravarty, Tobias Ackels, Cécile Piot, Bruna Almeida, Joana Carvalho, Alessandro Ciccarelli, Christopher J. Peddie, Neil Rogers, James Briscoe, François Guillemot, Andreas T. Schaefer, Leonor Saúde, Caetano Reis e Sousa

**Affiliations:** 1 Immunobiology Laboratory, The Francis Crick Institute, 1 Midland Road, London NW1 1AT, UK; 2 Instituto de Medicina Molecular, Faculdade de Medicina da Universidade de Lisboa, 1649-028 Lisboa, Portugal; 3 TechnoPhage, SA, Av. Prof. Egas Moniz, 1649-028 Lisboa, Portugal; 4 Bioinformatics and Biostatistics, The Francis Crick Institute, 1 Midland Road, London NW1 1AT, UK; 5 Sensory Circuits and Neurotechnology Laboratory, The Francis Crick Institute, 1 Midland Road, London NW1 1AT, UK; 6 Experimental Histopathology, The Francis Crick Institute, 1 Midland Road, London NW1 1AT, UK; 7 Advanced Light Microscopy, The Francis Crick Institute, 1 Midland Road, London NW1 1AT, UK; 8 Electron Microscopy, The Francis Crick Institute, 1 Midland Road, London NW1 1AT, UK; 9 Developmental Dynamic Laboratory, The Francis Crick Institute, 1 Midland Road, London NW1 1AT, UK; 10 Neural Stem Cell Biology Laboratory, The Francis Crick Institute, 1 Midland Road, London NW1 1AT, UK; 11 Department of Neuroscience, Physiology &Pharmacology, University College London, London, UK; 12 Instituto de Medicina Molecular e Instituto de Histologia e Biologia do Desenvolvimento, Faculdade de Medicina da Universidade de Lisboa, 1649-028 Lisboa, Portugal

**Keywords:** ependymal cells, neural stem cells, tissue repair, CLEC9A, dendritic cells

## Abstract

Cells with latent stem ability can contribute to mammalian tissue regeneration after damage. Whether the central nervous system (CNS) harbors such cells remains controversial. Here, we report that DNGR-1 lineage tracing in mice identifies an ependymal cell subset, wherein resides latent regenerative potential. We demonstrate that DNGR-1-lineage-traced ependymal cells arise early in embryogenesis (E11.5) and subsequently spread across the lining of cerebrospinal fluid (CSF)-filled compartments to form a contiguous sheet from the brain to the end of the spinal cord. In the steady state, these DNGR-1-traced cells are quiescent, committed to their ependymal cell fate, and do not contribute to neuronal or glial lineages. However, trans-differentiation can be induced in adult mice by CNS injury or *in vitro* by culture with suitable factors. Our findings highlight previously unappreciated ependymal cell heterogeneity and identify across the entire CNS an ependymal cell subset wherein resides damage-responsive neural stem cell potential.

## Highlights

• DNGR-1 is expressed early in mouse embryogenesis in a subset of ventricular progenitors
• DNGR-1 tracing shows that those progenitors give rise to a subset of ependymal cells
• DNGR-1-traced ependymal cells have latent regenerative potential
• DNGR-1-traced ependymal cells can be mobilized by local injury

## Graphical abstract

**Figure:**
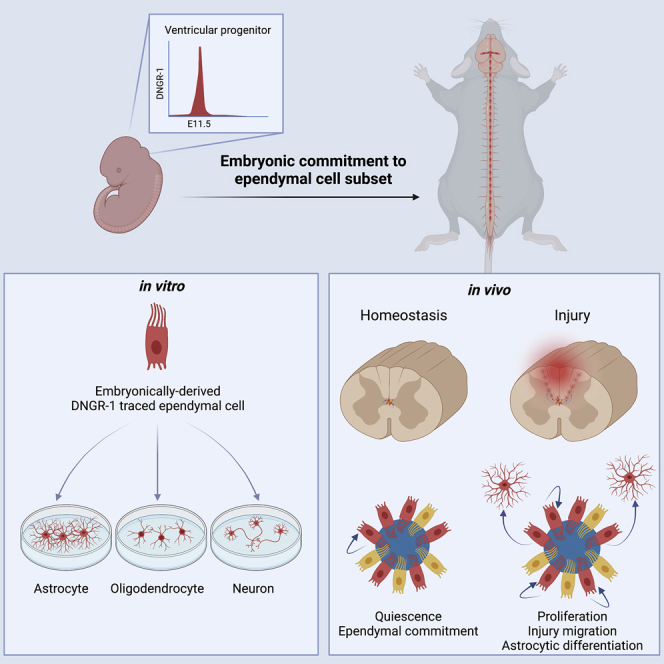

## Teaser

Frederico et al. show that embryonic DNGR-1 expression marks a population of ventricular progenitors committed to an ependymal cell subset yet endowed with damage-responsive neural stem cell potential in adulthood. Trans-differentiation can by induced *in vitro* with appropriate factors or *in vivo* after CNS injury.

## Introduction

Tissues such as the intestine or skin undergo constitutive turnover sustained by professional stem cells that proliferate and continuously differentiate into the cell types that make up the organ (Barker et al., 2007; Blanpain and Fuchs, 2006). Other tissues such as the pancreas or lung display lower cell turnover; however, this can be greatly accelerated after injury (Tata et al., 2013; Zhou and Melton, 2018). Injury-triggered tissue renewal programs differ from those in the steady state in that they do not always require a dedicated stem cell pool. This is best exemplified by the liver, which lacks a professional stem cell population yet is capable of marked regeneration following injury, mediated by both hepatocyte replication and the emergence of facultative stem cells (Raven et al., 2017; Tarlow et al., 2014). Such facultative or latent stem cells frequently originate from quiescent or fully differentiated cells, and their contribution to tissue regeneration in response to injury can even overshadow that of professional stem cells in tissues that contain the latter (Clevers and Watt, 2018; Post and Clevers, 2019). Latent stem cells are operationally defined by the ability to replace cells lost upon injury, and, therefore, their identification requires retrospective analysis. In mouse models, this can be achieved using genetic tools such as lineage tracing (a.k.a. fate mapping), which allows one to assess whether a given cell population contributes to other lineages before and after application of tissue stress (Kretzschmar and Watt, 2012).

The adult mammalian central nervous system (CNS) constitutes one of the least regenerative organs, although it contains professional stem cells that sustain neurogenesis throughout life (Doetsch et al., 1999; Gage, 2000; Seri et al., 2001; Spalding et al., 2013). These adult neural stem cell (NSC) populations inhabit two discrete brain niches, the subventricular zone (SVZ) and the dentate gyrus (DG), and generate neurons that integrate neuronal circuits in the olfactory bulb (OB) and the hippocampus, respectively (Obernier and Alvarez-Buylla, 2019). Although these NSC compartments have been reported to reactively upregulate neurogenesis in response to brain insult (Dash et al., 2001; Liu et al., 2017; Parent and Lowenstein, 2002), the newly generated neurons show limited survival and circuit integration (Quadrato et al., 2014). Furthermore, no equivalent stem cell compartment has been identified in the spinal cord. Thus, whether CNS resident professional stem cells significantly contribute to CNS repair remains unclear and the prognosis for CNS damage, including for traumatic spinal cord injury, remains poor. This has provided impetus for assessing whether the CNS possesses facultative stem cells that can be mobilized for tissue repair.

Ependymal cells have been postulated to constitute one such latent stem cell compartment. Ependymal cells form a ciliated epithelial sheet lining cerebrospinal fluid (CSF)-filled compartments in the CNS. Coordinated beating of their cilia promotes CSF flow from the brain ventricles through the central canal of the spinal cord. The motile ciliogenesis program is governed by the transcription factor Forkhead Box protein J1 (FoxJ1) (Yu et al., 2008), and some mouse lineage-tracing studies based on FoxJ1 promoter activity have suggested that ependymal cells or their progeny can trans-differentiate into astrocytes upon spinal cord injury (Barnabé-Heider et al., 2010; Carlén et al., 2009; Llorens-Bobadilla et al., 2020; Meletis et al., 2008; Sabelström et al., 2013). However, this has not been seen in other FoxJ1-lineage-tracing models (Muthusamy et al., 2014, 2018; Ren et al., 2017). Furthermore, FoxJ1 can be expressed by non-ependymal cells, casting uncertainty on the fidelity of FoxJ1-based lineage tracing as a proxy for ependymal origin (Beckervordersandforth et al., 2010; Devaraju et al., 2013; Jacquet et al., 2009). Finally, a distinct lineage-tracing approach based on αSMA expression has failed to reveal facultative stem cell ability in ependymal cells (Shah et al., 2018). Thus, it remains unclear whether ependymal cells possess latent stem cell ability and whether this property is perhaps confined to a sub-population of these seemingly homogeneous cells.

Here, we report that a subset of ependymal cells with latent stem cell potential can be defined in mice by its historical expression of DNGR-1, a receptor hitherto only found in cells of the immune system. DNGR-1-lineage-traced ependymal cells arise very early during embryogenesis to form a contiguous layer that extends from the brain ventricles through the entire spinal cord. Although these cells remain quiescent during homeostasis, they can undergo extensive proliferation and differentiation in response to spinal cord or brain damage. Therapeutic approaches aimed at harnessing their regenerative potential may hold promise in treating CNS injuries.

## Results

### DNGR-1 lineage tracing defines a non-dendritic cell compartment lining the brain ventricular system and spinal cord central canal

**Figure 1:**
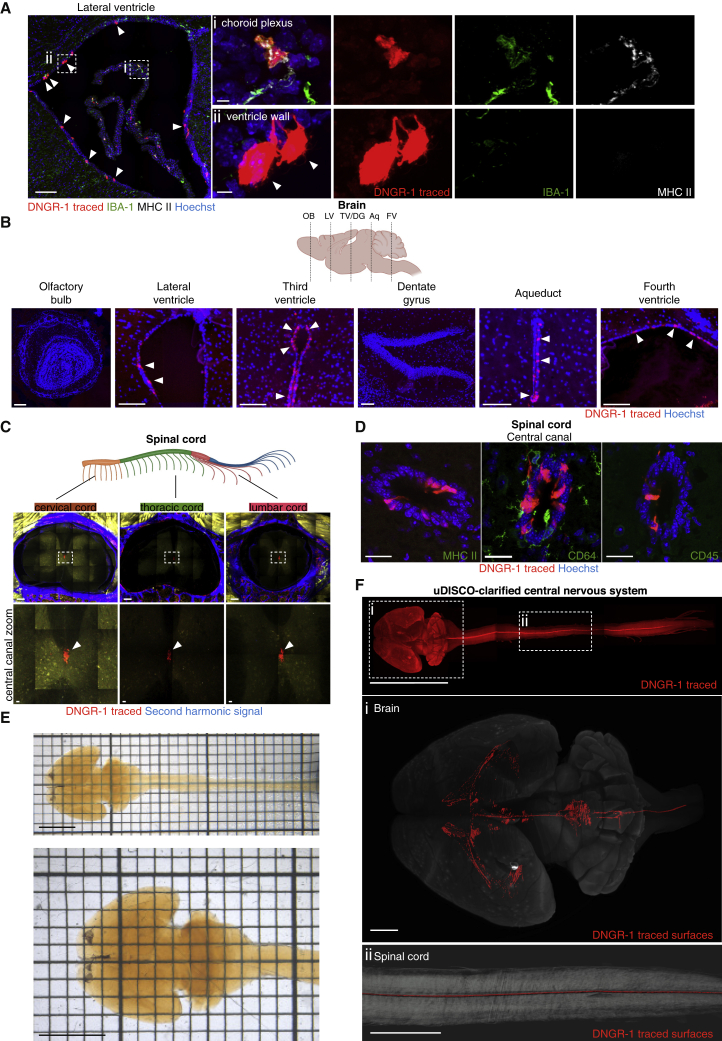
DNGR-1 lineage tracing marks non-cDCs lining the brain ventricular system and the spinal cord central canal (A) Brain cryosection from a *Clec9a*^*Cre*^*Rosa*^*LSLtdTomato*^ animal stained with antibodies against IBA-1 (green), MHC II (white); zooms: (Ai) choroid plexus (Aii) ventricle wall. Arrowheads indicate DNGR-1-traced cells (red) in the ventricle wall lacking IBA-1 and MHC II staining. (B) Brain coronal cuts showing DNGR-1-traced cells (red, arrowheads) in the ventricle walls of all CSF-filled compartments. No DNGR-1-traced cells (red) were observed in olfactory bulbs or dentate gyrus. OB, olfactory bulb; LV, lateral ventricle; TV, third ventricle; DG, dentate gyrus; Aq, aqueduct; FV, fourth ventricle. (C) Multiphoton image of vibratome spinal cord sections showing DNGR-1-traced cells (red, arrowheads) around the central canal in different rostral-caudal levels. Second harmonic signal, blue. (D) Spinal cord cryosections labeled with antibodies against MHC II, CD64, or CD45 (all green). (E and F) Optically clarified (E) and light-sheet imaged (F) whole-CNS with DNGR-1-traced cells (red) lining the entire ventricular compartment. Bottom, zoomed view of indicated areas (Fi) brain (Fii) spinal cord. Scale bars, 5 mm (E), 1 mm (F), 100 μm (A–D and zoom F), and 20 μm (zooms A and C). Sections from at least 3 animals were analyzed per experiment.

Dendritic cells are an important type of bone marrow-derived myeloid cell involved in immune regulation. DNGR-1 (also known as CLEC9A) is expressed by the conventional dendritic cell (cDC) subtype 1 (cDC1), as well as by cDC-committed progenitors in the bone marrow (Schraml et al., 2013). Taking advantage of the latter finding, we previously generated a DNGR-1 lineage tracer mouse (*Clec9a*^*Cre*^*Rosa*^*LSLtdTomato*^) in which the history of DNGR-1 expression defines the entire cDC lineage (Schraml et al., 2013). We initially set out to use *Clec9a*^*Cre*^*Rosa^LSLtdTomato^* lineage tracing to identify and study cDCs in the CNS. Immunohistological analysis of brains from adult mice readily revealed the presence of tdTomato^+^ cells displaying morphology and markers of cDCs (MHC class II [MHC II^+^] and IBA-1^+^) (Figure 1Ai). These DNGR-1-traced cDCs could be found in ample numbers in the meninges (data not shown) and choroid plexi (Figure 1Ai), as expected (Anandasabapathy et al., 2011; Quintana et al., 2015). Surprisingly, we also noticed the presence of DNGR-1-traced cells that lacked expression of canonical cDC markers lining the brain ventricles (tdTomato^+^, MHC II^−^, and IBA-1^−^; Figure 1Aii). These cells are henceforth termed non-cDC, DNGR-1-traced cells.

The brain ventricular system comprises four cisternae and extends caudally into the central canal of the spinal cord (Korzh, 2018). Serial brain sectioning demonstrated non-cDC, DNGR-1-traced cells embedded in the lining of all four ventricles (Figure 1B). Multiphoton imaging of different rostral-caudal regions of spinal cord demonstrated that non-cDC, DNGR-1-traced cells also lined the central canal (Figure 1C). As for the cells in brain ventricles, labeled cells in the central canal lacked expression of MHC II and CD64 (cDC and macrophage markers) or the pan-leucocyte marker, CD45 (Figure 1D). To assess if non-cDC, DNGR-1-traced cells formed a contiguous layer, we dissected the entire CNS from a DNGR-1-lineage-traced mouse and subjected it to optical clarification and light-sheet imaging (Figure 1E; Videos S1, S2, and S3). Non-cDC, DNGR-1-traced cells were evenly distributed from the brain ventricles to the sacral portion of the spinal cord in a pattern that corresponded to the boundaries of the CSF-filled network (Figure 1E; Video S3). The cells were also found in a DNGR-1 lineage tracer mouse strain made as a BAC transgenic (Schraml et al., 2013), excluding the possibility that labeling somehow results from disruption of the endogenous *Clec9a* locus (data not shown). Thus, in addition to marking *bona fide* cDCs in mice, DNGR-1 lineage tracing reveals a CNS cell population inhabiting the lining of the brain ventricular system and the spinal cord central canal.

Video S1. Light-sheet microscopy video of whole CNS from a *Clec9a*^*Cre*^*Rosa*^*LSLtdTomato*^ mouse after uDISCO optical clarification, related to Figure 1 Continuum of DNGR-1-traced cells (red) from the sacral part of the spinal cord to the brain ventricular compartment.

Video S2. Confocal microscopy video of uDISCO optically clarified spinal cord from a *Clec9a*^*Cre*^*Rosa*^*LSLtdTomato*^ mouse, related to Figure 1 Dorsal perspective of the spinal cord counterstained with Hoechst (blue) demonstrating DNGR-1-traced cells (red) lining the central canal.

Video S3. Surface representation of a light-sheet microscopy video of a uDISCO clarified brain from a *Clec9a*^*Cre*^*Rosa*^*LSLtdTomato*^ mouse, related to Figure 1 Brain DNGR-1-traced surfaces (red) lining the ventricular compartment. Surfaces were calculated with Imaris software. Brain contour was defined by tissue autofluorescence and DNGR-1-traced surfaces were defined by bright tdTomato fluorescence.

### Non-cDC, DNGR-1-traced cells are unrelated to cDCs

**Figure 2:**
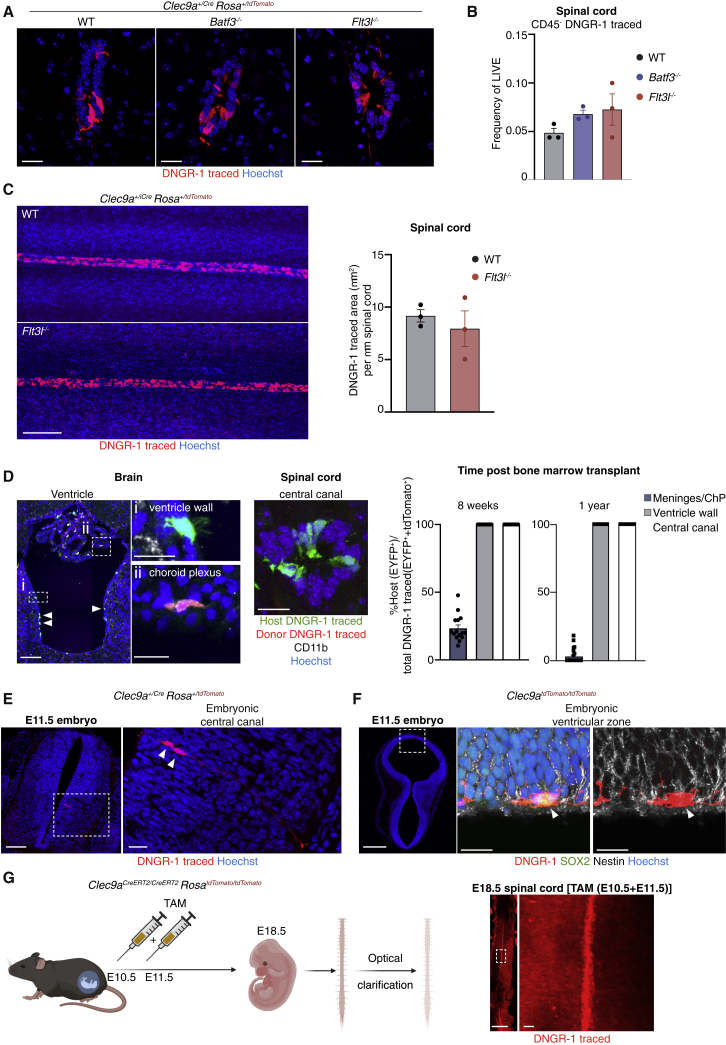
Non-cDC, DNGR-1-traced cells are embryonically derived (A) Spinal cord central canal cryosections of *Clec9a*^*Cre*^*Rosa*^*LSLtdTomato*^ animals lacking *Batf3* or *Flt3l*. (B) Flow cytometric quantification of CD45^−^ DNGR-1-traced cells in spinal cords of *Clec9a*^*Cre*^*Rosa*^*LSLtdTomato*^ animals lacking *Batf3* or *Flt3l*. (C) Maximum intensity projection of optically cleared spinal cords of *Clec9a*^*Cre*^*Rosa*^*LSLtdTomato*^ animals lacking *Flt3l* or not (WT). Right, quantification of DNGR-1-traced area (μm^2^) per μm of spinal cord. Each dot represents one animal. (D) Brain and spinal cord cryosections of irradiated *Clec9a*^*Cre*^*Rosa*^*LSLEYFP*^ mouse 8 weeks after transplantation with *Clec9a*^*Cre*^*Rosa*^*LSLtdTomato*^ bone marrow labeled with anti-CD11b (white). Host-derived radioresistant DNGR-1-traced cells (EYFP^+^, green) line the brain ventricle (white arrowheads) and the central canal of the spinal cord. (Di) Multi-ciliated host-derived DNGR-1-traced ependymal cell (green) in the ventricle wall; (Dii) CD11b^+^ (white) donor-derived hematopoietic DNGR-1-traced cell (red) in the choroid plexus. Right. Quantification of sections across 3–5 animals. ChP, choroid plexus. (E) Spinal cord cryosection from an E11.5 *Clec9a*^*Cre*^*Rosa*^*LSLtdTomato*^ embryo showing two DNGR-1-traced cells (red, arrowheads). DNGR-1-traced cells were not ciliated at this time point. Right, zoomed view. (F) Brain cryosection from an E11.5 *Clec9a*^*tdTomato*^ embryo showing a DNGR-1-expressing cell embedded in the ventricular wall. Right, zoomed view and staining with SOX2 (green), nestin (white), and Hoechst (blue). (G) *In utero* induction of lineage tracing. Right, wholemount optically clarified spinal cord from *Clec9a*^*CreERT2*^*Rosa*^*LSLtdTomato*^ E18.5 embryo after *in utero* exposure to tamoxifen at E10.5 and E11.5. Zoomed-in area shows DNGR-1 tracing (red) of the ependymal cell layer. Scale bars, 500 μm (E, spinal cord, F, and G), 100 μm (A, C, D, and zooms E), and 20 μm (zoom D, F, and G). At least 3 animals or were analyzed per experiment. 1 embryo was analyzed per experiment.

The absence of canonical markers argued that non-cDC, DNGR-1-traced cells were unrelated to their DNGR-1-traced cDC counterparts. To confirm this, we assessed the frequency of both DNGR-1-traced cellular populations in DNGR-1-traced animals crossed to mice deficient in BATF3 (a transcription factor required for the development of cDC1s) (Hildner et al., 2008) or FLT3L (a growth factor essential for development and survival of the entire cDC lineage) (Cabeza-Cabrerizo et al., 2021). As expected, numbers of DNGR-1-traced cDC1s and cDCs were severely reduced in mice lacking *Batf3* and *Flt3l*, respectively (Figure S1A). In contrast, the frequency of non-cDC, DNGR-1-traced cells remained unaltered (Figures 2A and 2B). Light-sheet imaging of wholemount-clarified spinal cords confirmed that the absence of FLT3L did not affect the number, localization, or anatomical distribution of the non-cDC, DNGR-1-traced compartment (Figure 2C; Video S4).

Video S4. Confocal microscopy video of uDISCO clarified spinal cords from *Clec9a*^*Cre*^*Rosa*^*LSLtdTomato*^ and *Clec9a*^*Cre*^*Rosa*^*LSLtdTomato*^*Flt3l*^*−/−*^ mice, related to Figure 2 Side-by-side comparison of spinal cords from *Clec9a*^*Cre*^*Rosa*^*LSLtdTomato*^ and its cDC deficient counterpart (*Clec9a*^*Cre*^*Rosa*^*LSLtdTomato*^*Flt3l*^*−/−*^) showing no reduction of DNGR-1- traced cells (red) lining the central canal of the spinal cord. Nuclei were counterstained with Hoechst (blue).

We also generated bone marrow chimeras in which irradiated DNGR-1 EYFP^+^ lineage-traced mice were reconstituted with DNGR-1 tdTomato^+^-lineage-traced bone marrow (Figure S1B). In this experimental system, tdTomato expression reports on bone marrow origin, whereas EYFP fluorescence reports host-derived radioresistant DNGR-1-traced cells. Consistent with hematopoietic origin, the vast majority of DNGR-1-traced cDCs in the meninges and choroid plexus (ChP) was tdTomato^+^ (Figure 2D). In contrast, 100% of nonDC, DNGR-1-traced cells lining the brain ventricles and the spinal cord central canal were EYFP^+^ (Figure 2D), reflecting host origin. Analysis more than 1 year post-transplantation (Figure S1B) revealed that meningeal and ChP DNGR-1-traced cDCs had been completely replenished by donor bone marrow-derived cells (tdTomato^+^), but even at this time point, their non-cDC, DNGR-1-traced counterpart remained 100% EYFP^+^ (Figures 2D and S1B). Thus, non-cDC, DNGR-1-traced cells do not originate from bone marrow-derived hematopoietic precursors.

### Non-cDC, DNGR-1-traced cells emerge from DNGR-1-expressing ventricular progenitors at E11.5

Further underscoring their non-hematopoietic origin, the first non-cDC, DNGR-1-traced cells emerged at embryonic day 11.5 (E11.5), a developmental stage that precedes the advent of definitive hematopoiesis (Golub and Cumano, 2013). Analysis of optically clarified E11.5 DNGR-1-traced embryos (Figure S1C) complemented by immunohistological analysis (Figure 2E) demonstrated that some of these cells were already integrated in the walls of the brain ventricles and in the central canal of the spinal cord. As this developmental time point marked the emergence of DNGR-1-traced cells, it suggested that it also marked the inception of DNGR-1 lineage tracing by DNGR-1-expressing cells. To assess this, we generated a DNGR-1 reporter mouse by introducing tdTomato into the Clec9a locus (*Clec9a*^*tdTomato*^). We first validated that *Clec9a*-driven tdTomato is expressed in DNGR-1-positive cells by examining the cDC lineage (cDC1s, which are DNGR-1^+^, were nearly 100% tdTomato^+^, whereas cDC2, which do not express DNGR-1, were <1% labeled) (Figure S1D). Analysis of E11.5 DNGR-1 reporter embryos showed rare DNGR-1^+^ cells embedded in the ventricular zone, which displayed concurrent expression of the progenitor marker SOX2 but not the neuroepithelial marker nestin (Figure 2F). In contrast, no DNGR-1^+^ cells were ever found in the ependymal layer of adult *Clec9a*^*tdTomato*^ reporter mice (Figure S1E), indicating that expression is confined to embryonic stages. To formally test whether the rare DNGR-1-expressing cell population at E11.5 could serve as the embryonic progenitors of non-cDC, DNGR-1-traced cells, we further developed a tamoxifen-inducible DNGR-1-lineage-tracing model (*Clec9a*^*CreERT2*^*Rosa^LSLtdTomato^*). Validation of this transgenic strain was again carried out by monitoring labeling of the cDC lineage upon tamoxifen administration to adult animals, which led to the expected robust labeling of the cDC1 compartment and lower cDC2 marking (cDC2 lack DNGR-1 expression but arise from DNGR-1-positive progenitors [Schraml et al., 2013; Figure S1F]). *In utero* induction of DNGR-1 lineage tracing 1 day prior to and at the time when the first DNGR-1-expressing cells are detected (E10.5 + E11.5) and analysis of wholemount optically clarified spinal cords 1 week later (E18.5) recapitulated the spinal cord-labeling pattern observed in constitutive DNGR-1-lineage-tracing adult mice (Figure 2G). In contrast, such labeling was never seen in *Clec9a*^*CreERT2*^*Rosa^LSLtdTomato^* mice treated with tamoxifen after birth (Figure S1G). Thus, transient DNGR-1 expression marks a population of ventricular embryonic progenitors fated to originate the non-cDC, DNGR-1-traced compartment found in the adult CNS.

### The non-cDC, DNGR-1-traced compartment corresponds to ependymal cells

**Figure 3:**
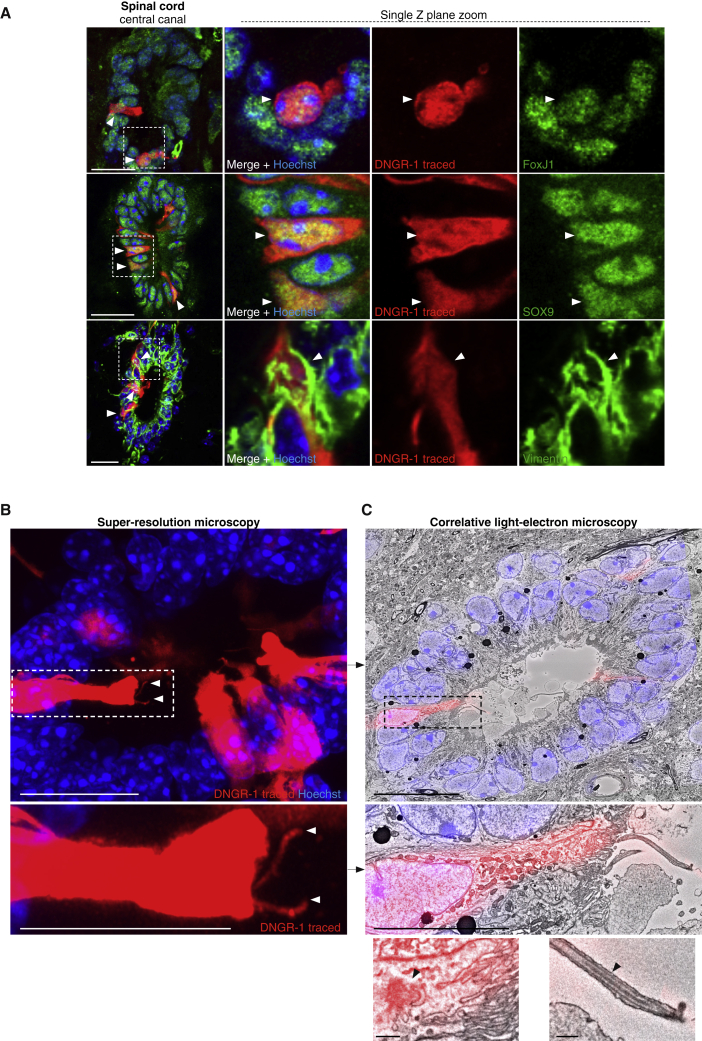
Non-cDC, DNGR-1-traced cells are ependymal cells (A) Spinal cord central canal cryosections from *Clec9a*^*Cre*^*Rosa*^*LSLtdTomato*^ mice labeled with antibodies against FoxJ1, SOX9, or vimentin (green). Zoomed-in panels show single optical Z slice of indicated areas. Arrowheads indicate DNGR-1-traced cells co-stained with the respective marker. (B) Airyscan super-resolution of vibratome section from a DNGR-1-traced spinal cord central canal. Arrowheads in zoomed view indicate two cilia emerging from DNGR-1-traced ependymal cells. (C) Composite of the super-resolution image shown in (B) on respective electron micrograph. Bottom, zoomed view of indicated area. Below left, basal body (arrowhead) and; below right, cilium displaying a 9 + 2 axoneme structure (arrowhead). Scale bars, 20 μm (A–C), 10 μm (zooms B and C), and 0.5 μm (detailed views of C). Representative images of 2 animals analyzed.

To assign an identity to non-cDC, DNGR-1-traced cells, we focused on the known cellular composition of the spinal cord. Non-cDC, DNGR-1-traced cells lacked expression of GFAP, NeuN, PKD2L1, NG2, or PDGFrβ, indicating that they were not astrocytes, neurons, CSF-contacting neurons, oligodendrocyte progenitors, or pericytes, respectively (Figure S2). However, non-cDC, DNGR-1-traced cells were uniformly positive for FoxJ1 and expressed SOX9 and vimentin (Figure 3A), consistent with being ependymal cells. Using correlative light (super-resolution) and electron (serial block-face) microscopy (Figures 3B and 3C), most non-cDC, DNGR-1-traced cells in the spinal cord central canal were found to have a simple/pseudostratified columnar epithelial cell shape (Figure S3A) and to possess two cilia (Figure 3B; Video S5), which displayed a 9+2 axoneme structure (Figures 3C and S3B), consistent with motile nature. Both cilia averaged a length of 11.86 ± 2.85 μm (Figure S3C) and could be further tracked through the image volume to their respective basal bodies (Figures 3C and S3D; Video S6). Thus, immunophenotypic and ultrastructural characterization identifies non-cDC, DNGR-1-traced cells in the CNS as ciliated ependymal cells.

Video S5. Super-resolution airyscan video of the central canal of the spinal cord from a *Clec9a*^*Cre*^*Rosa*^*LSLtdTomato*^ mouse, related to Figure 3 Representative vibratome section imaged with the airyscan super-resolution module which reveals two cilia emerging from ependymal DNGR-1-traced cells (red). Nuclei were counterstained with Hoechst (blue).

Video S6. Correlative light-electron microscopy video tracking cilia of DNGR-1-traced ependymal cells, related to Figure 3 Tracking of an individual cilium belonging to an DNGR-1-traced ependymal cell (red), from its central canal extension to its respective basal body.

### DNGR-1-traced ependymal cells exist along a differentiation continuum

**Figure 4:**
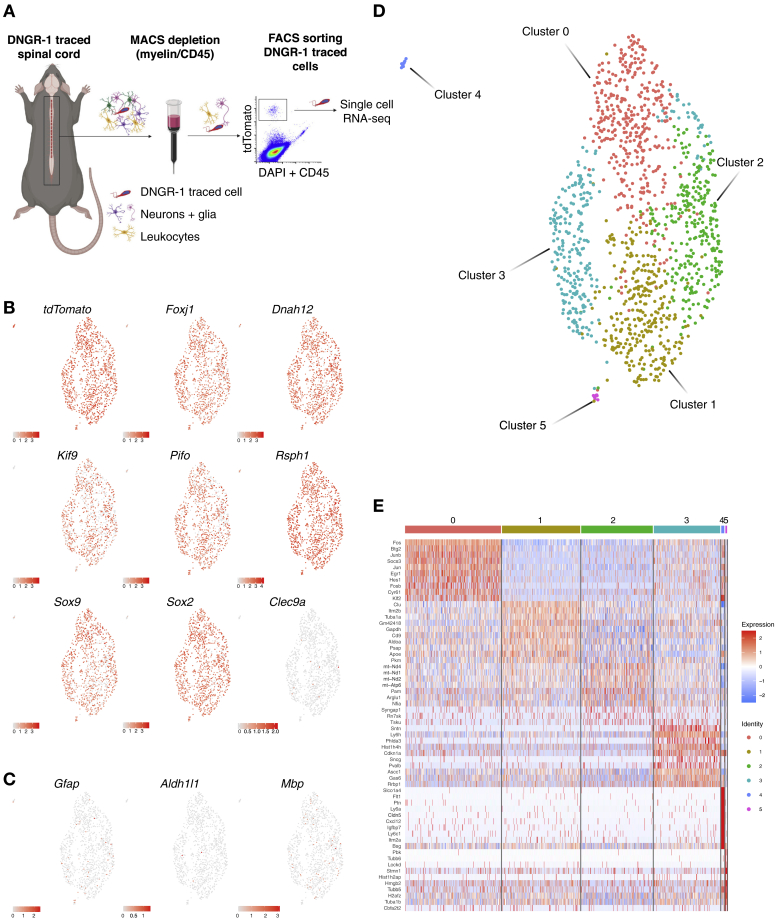
Single-cell RNA sequencing of non-cDC, DNGR-1-traced cells (A) Purification of non-cDC, DNGR-1-traced cells. (n = 14 pooled spinal cords). (B and C) (B) Feature plots showing projection of *tdTomato*, ependymal cell identity, or *Clec9a* and (C) glial cell transcripts on the UMAP space. Low expression (gray) and high expression (red). (D) UMAP embedding of 1,241 DNGR-1-traced cells and unsupervised clustering showing high compactness of DNGR-1-traced ependymal cells. (E) Heatmap representation of the top-10 differentially expressed genes defining each cluster. Low expression (blue) and high expression (red).

We carried out single-cell RNA sequencing (scRNA-seq) of CD45^−^ DNGR-1-traced cells isolated from spinal cords (Figure 4A). After initial quality control, data from 1,241 cells were analyzed. Mapping of tdTomato reads onto the dataset confirmed that the cells originated from the DNGR-1-traced compartment (Figure 4B). In agreement with being ependymal cells, the vast majority expressed the genes encoding the master regulator of motile cilia (*Foxj1*), the structural components of cilia, such as dynein (*Dnah12*) and kinesin (*Kif9*) motors, the basal body that attaches cilia to the cell body (*Pifo*) and the radial spokes (*Rsph1*), as well as the transcription factors *Sox9* and *Sox2* (Figure 4B). Conversely, markers of astrocytes (*Gfap*, *Aldh1l1*) or oligodendrocytes (*Mbp*) were expressed at low levels by a negligible number of cells (Figure 4C). Other markers of oligodendrocytes (*Olig1*, *Olig2*, *Mog*, and *Nkx2-2*), oligodendrocyte progenitors (*Sox10* and *Pdgfra*), or neuroblasts (*Eomes* and *Tbr1*) were completely absent from the dataset. Indeed, based on their global gene expression profile, 97% of the cells analyzed (1,204 cells of 1,241) matched the ependymal cell identity defined by the CNS single-cell reference atlas (Zeisel et al., 2018). *Clec9a* (which encodes DNGR-1) was not appreciably detected (Figure 4B; 3 cells of 1,241), consistent with the fact that it is only expressed in the embryonic precursors of the traced cells (see above).

Analysis by UMAP dimensionality reduction and unsupervised clustering allowed sub-grouping of DNGR-1-traced ependymal cells into 6 clusters (Figures 4D and 4E). The latter did not correspond to groups of cells occupying distinct dorsal-ventral or rostral-caudal positions, as transcription factors governing such positioning (i.e., ZEB1, PAX6 and Hox-A5, Hox-A9, and Hox-A10, respectively) (Ghazale et al., 2019) were found across all clusters (data not shown). Cluster 4 (12 cells of 1,241) stood out by the expression of hallmark genes of vascular endothelial cells such as *Cldn5, Flt1*, and *Pecam1* and concurrent absence/low expression of ependymal cell signature transcripts, including *Foxj1*, and was excluded from further analysis. Apart from cluster 4, the remaining clusters displayed high compactness within the UMAP space, suggesting that DNGR-1-traced ependymal cells are relatively homogeneous and that clusters likely represent cellular states along a lineage continuum rather than discrete subsets.

### DNGR-1-traced ependymal cells display neural stem cell properties *in vitro*

**Figure 5:**
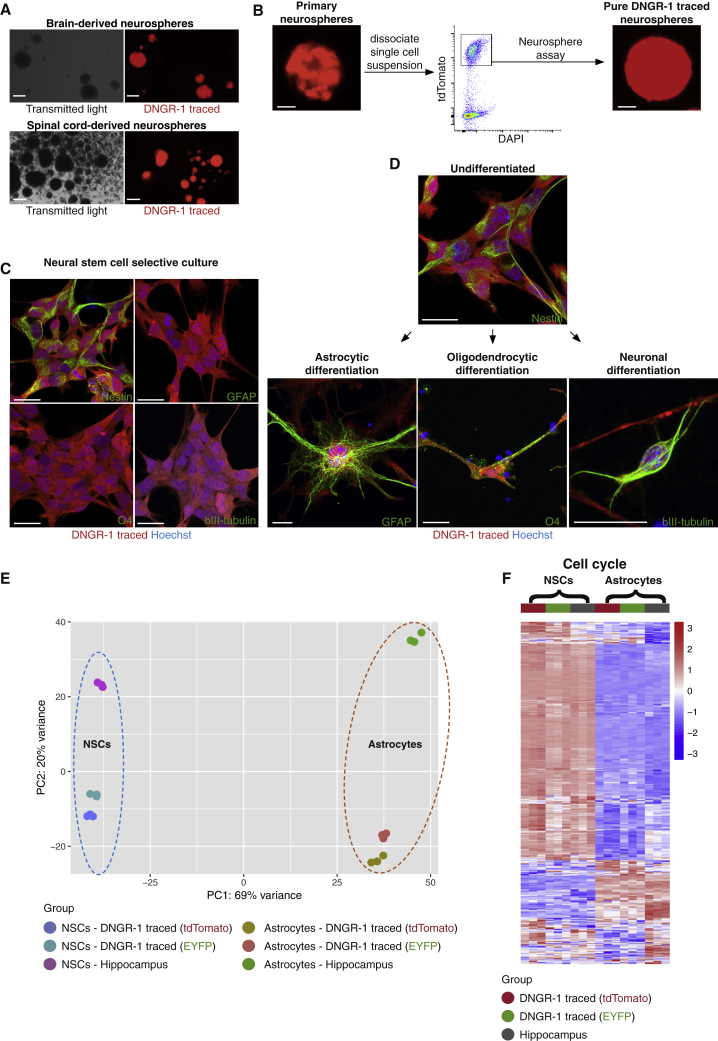
DNGR-1-traced ependymal cells display neural stem cell properties *in vitro* (A) Representative images from primary neurosphere cultures grown from brain or spinal cord of *Clec9a*^*Cre*^*Rosa*^*LSLtdTomato*^ mice. (B) Strategy for isolation of pure DNGR-1-traced neurospheres (tdTomato^+^). (C and D) (C) Immunofluorescence of pure DNGR-1-traced neurosphere-derived cells (red) grown in adherence in the absence (C) or presence (D) of differentiation promoting factors and stained with antibodies against nestin, GFAP, O4, and βIII-tubulin (all green). (E) Principal component analysis showing clustering of hippocampi-derived or DNGR-1-traced NSCs and their respective differentiated astrocytes. Each dot represents a biological replicate. (F) Heatmap of differential expressed genes involved in cell cycle between NSC and astrocyte cell fates derived from the hippocampus or from DNGR-1-traced cells. Scale bars, 200 μm (A), 100 μm (B), and 20 μm (C and D). Representative images of least 3 independent experiments.

Neurosphere assays allow for selective expansion of stem and progenitor cells *in vitro* (Reynolds and Weiss, 1992). Interestingly, preparations from brains and spinal cords of DNGR-1-lineage-traced mice gave rise to neurospheres containing DNGR-1-traced cells (Figure 5A). This was not due to upregulation of DNGR-1 *ex vivo* as no DNGR-1-traced neurospheres could be observed when grown from brains or spinal cords of *Clec9a*^*CreERT2*^*Rosa^LSLtdTomato^* mice in the presence of 4-hydroxytamoxifen (Z-4OH-TAM) (Figure S4A). In contrast, Z-4OH-TAM-treated *in vitro* cDC cultures (Helft et al., 2015) grown from bone marrow of these animals yielded robust labeling of cDC1 (>98%), controlling for the sensitivity of the system (Figure S4B).

On closer inspection, primary neurospheres contained both DNGR-1-traced and non-traced cells (Figure 5B). Primary neurospheres can be composed of stem and progenitor cells, the former being distinguished by their ability to self-renew over an extended period of time. Notably, when we FACS-sorted tdTomato^+^ cells from primary neurospheres and re-plated them, we were able to re-generate neurospheres that now contained 100% tdTomato^+^ cells and that could be grown and passaged repeatedly (Figure 5B). Thus, DNGR-1-traced cells in neurospheres are capable of prolonged self-renewal.

The proportion of actual NSCs within neurospheres is low (Reynolds and Rietze, 2005) but can be increased by adherent culture in NSC-favoring conditions (Conti et al., 2005; Pollard et al., 2006). In such cultures, neurosphere-derived DNGR-1-traced cells could be passaged for more than 6 months without cell loss and expressed the NSC marker nestin but not markers of differentiated CNS cells (Figure 5C). Remarkably, upon addition of suitable factors, the DNGR-1-traced cell cultures could be coaxed to differentiate into astrocytes, oligodendrocytes, or immature neurons (Figures 5D and S4C). We compared by bulk RNA-seq DNGR-1-traced cells and *bona fide* professional NSCs (isolated from the hippocampus rather than subventricular zone to avoid any possible contamination of the latter with ependymal layer), as well as their respective *in vitro* differentiated astrocyte progenies (Figures S4D and S4E). In principal component and cluster analysis, DNGR-1-traced NSCs (generated from two independent tracer mouse strains) clustered together with hippocampal NSCs both before and after differentiation, indicating that most of the variance was attributable to the differentiation process and not the cellular origin (Figure 5E). In their undifferentiated state, the similarity between the two NSC populations was also noticeable at the level of expression of genes involved in cell cycle, consistent with the ability of the cells to expand *in vitro* (Figure 5F). Thus, DNGR-1-traced ependymal cells display properties of stem cells when propagated and differentiated *in vitro* and are remarkably similar to *bona fide* NSCs under these culture conditions.

### CNS injury induces differentiation of DNGR-1-traced ependymal cells

**Figure 6:**
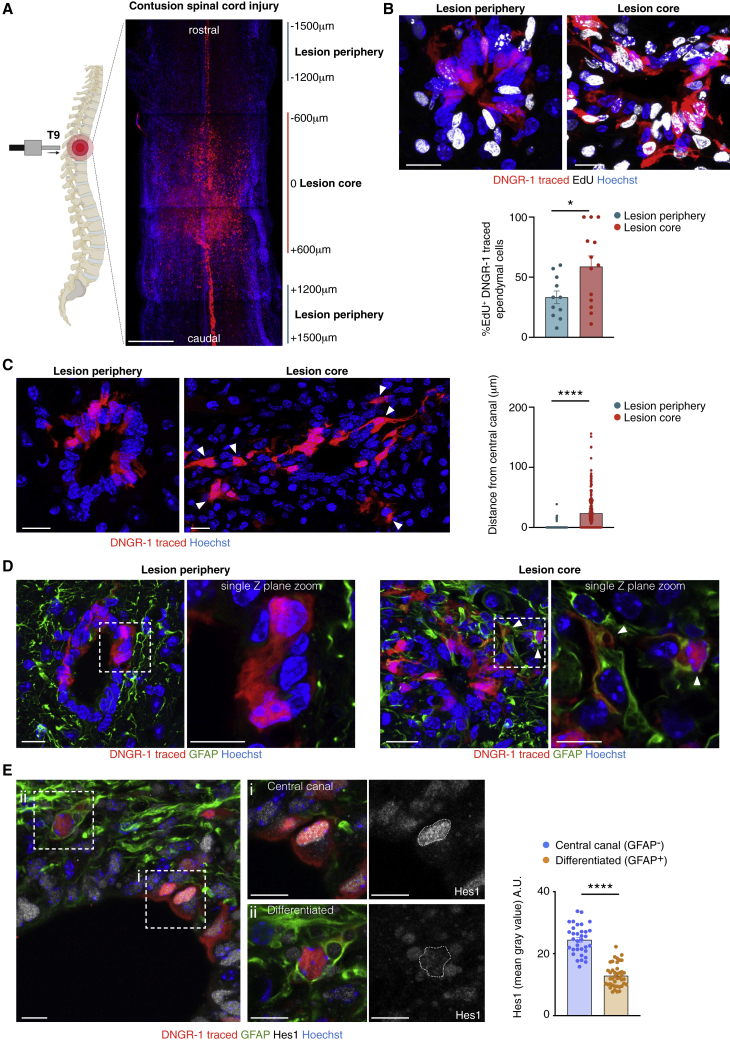
Mobilization and differentiation of DNGR-1-traced ependymal cells after injury (A) Maximum intensity projection of optically clarified spinal cord from an injured *Clec9a*^*Cre*^*Rosa*^*LSLtdTomato*^ mouse. T9 refers to injured vertebra. Lesion coordinates showed result from white matter sparring determination (Figure S5B). Lesion periphery = −1,500–−1,200 μm rostral and +1,200–+1,500 μm caudal; lesion core = −600 μm rostral to +600 μm caudal. (B–E) Spinal cord cryosections *from Clec9a*^*Cre*^*Rosa*^*LSLtdTomato*^ mice, 7 days post injury, showing: (B) EdU (white) incorporation by DNGR-1-traced ependymal cells (red) in the the lesion periphery or lesion core. Below, quantification in both sites. Each dot represents one quantified image. (C) Proximity of DNGR-1-traced cells to the central canal in the lesion periphery or core. Arrowheads show DNGR-1-traced cells displaced from the central canal. Right, quantification of distance (μm) of DNGR-1-traced cells from the central canal. Each dot represents one cell. (D) Staining with the astrocytic marker GFAP (green) in the central canal of the lesion periphery (left) or core (right). Arrowheads, GFAP^+^DNGR-1-traced cells. (E) Spinal cord central canal cryosection from *Clec9a*^*Cre*^*Rosa*^*LSLtdTomato*^ mice 21 days post injury stained labeled with anti-GFAP(green) and anti-Hes1 (white) antibodies. Right, zooms of (Ei) central canal or (Eii) peri-ependymal region. Right, quantification of Hes1 expression (a.u., arbitrary units). Error bars (SEM). Scale bars, 400 μm (A), 20 μm (C and D), 15 μm (B), and 10 μm (E). At least 3 animals were quantified per experiment. Groups were compared using unpaired t test. ^∗^p < 0.05, ^∗∗∗∗^p < 0.0001.

We assessed whether the differentiation and self-renewal potential of DNGR-1-traced ependymal cells could also be revealed *in vivo*. A fraction of DNGR-1-traced ependymal cells incorporated EdU over a 30-day period (Figure S5A), consistent with homeostatic proliferation. However, as mentioned, we did not find labeled neurons or glia in either spinal cord (Figure S2) or brain (Figure 1B) of DNGR-1-lineage-tracing mice, indicating lack of contribution to neuronal or glial lineages in the steady state. To determine whether this could be altered by injury, we subjected DNGR-1-lineage tracer mice to spinal cord contusion (Figure 6A). Histological examination identified an injury core comprising roughly 1,200 μm (Figure S5B) that extended symmetrically from the lesion epicentre (−600 μm rostral/+600 μm caudal). Rare DNGR-1-traced cDCs infiltrating the lesion could be distinguished from their traced ependymal cell counterparts by CD45 expression and dimmer tdTomato expression (Figure S5C). Light-sheet imaging revealed that the distribution pattern of DNGR-1-traced ependymal cells (tdTomato^bright^) remained unchanged in regions peripheral to injury but was significantly altered in the lesion core (Figure 6A; Video S7). There, DNGR-1-traced ependymal cells could be seen to have expanded and moved away from the central canal. Expansion appeared to be due to proliferation as EdU incorporation by DNGR-1-traced cells increased to approximately 60% in the lesion core (reaching 100% in some sections), compared with 30% in the lesion periphery (Figure 6B) 7 days after injury. Closer examination revealed that EdU incorporation correlated with displacement away from the central canal and acquisition of altered morphology (Figure 6C). Notably, a fraction of these displaced cells in the vicinity of the injury acquired GFAP immunoreactivity, consistent with astrocytic differentiation (Figure 6D). We did not observe differentiation into neurons or oligodendrocytes (Figure S5D). DNGR-1-traced cells away from the lesion epicenter were never found to express GFAP (Figure 6D), retained their usual morphology, and remained confined to the ependymal layer of the central canal (Figures 6A, 6C, and 6D), as suggested by the light-sheet images (Figure 6A; Video S7). We further tested a model of brain ventricular injury. Similar to spinal cord contusion, DNGR-1-lineage-traced ependymal cells lining the ventricles also reacted to local injury with morphological changes and migration toward the damage site, where some cells became GFAP^+^ (Figure S5E).

**Figure 7:**
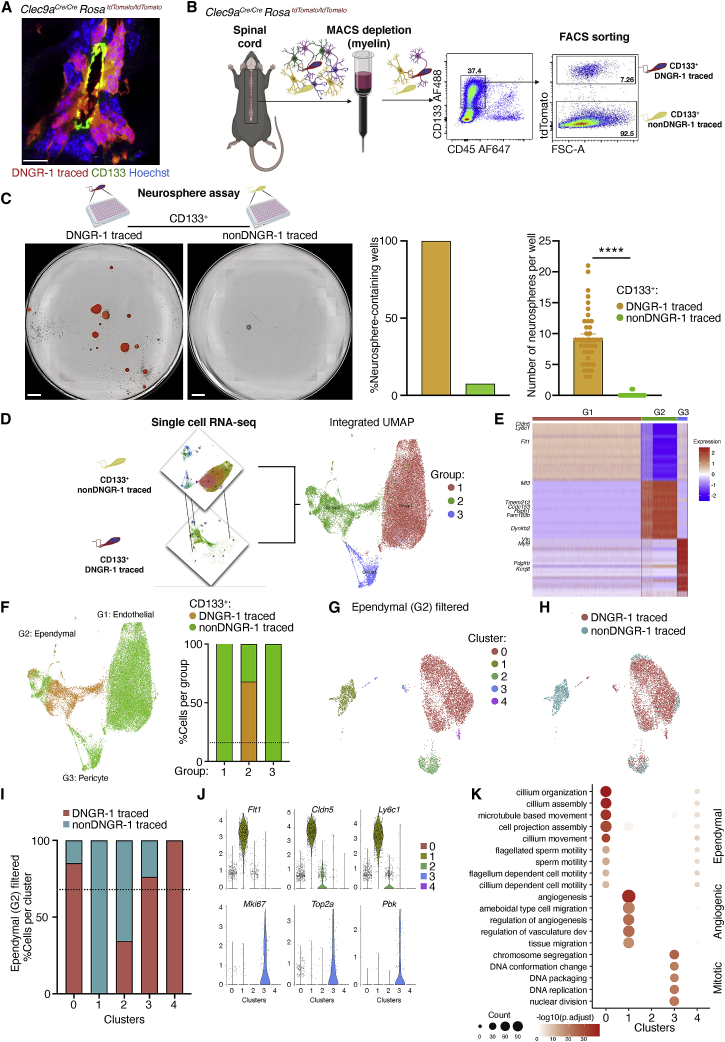
DNGR-1-tracing marks a distinct class of ependymal cells, wherein resides latent stem cell ability (A) Spinal cord central canal cryosection from a *Clec9a*^*Cre/Cre*^*Rosa*^*LSLtdTomato/LSLtdTomato*^ mouse labeled with anti-CD133 (green). CD133 is expressed apically and marks all ependymal cells (traced and untraced). (B) FACS-sorting of CD133^+^ DNGR-1-traced and CD133^+^ non-traced cells. (C) Neurosphere assay of CD133^+^ DNGR-1-traced and CD133^+^ non-traced cells from 4 pooled spinal cords. Right, quantification of neurosphere-containing wells and number of neurospheres per well from each fraction. Data are from two independent experiments. Error bars (SEM). Groups were compared using unpaired t test. *^∗∗∗∗^*p < 0.0001. (D) Dataset integration and UMAP of CD133^+^ DNGR-1-traced cells and CD133^+^ non-traced cells into 3 groups. (E) Heatmap of the top 20 differentially expressed genes in each group from (D). (F) UMAP representation by cellular provenance and quantification of each cellular fraction per group. Orange, CD133^+^ DNGR-1-traced cells; Green, CD133^+^ non-traced cells. 97.89% of CD133^+^ DNGR-1-traced cells were unbiasedly assigned to ependymal cell group (group 2). The remaining CD133^+^ DNGR-1-traced cells were assigned to groups 1 (1.89%) and 3 (0.23%). Dashed line represents the proportion of CD133^+^ DNGR-1-traced cells within the UMAP space (15.95%—4,825 of 30,326 cells). (G) UMAP of cells from group 2 (from F). (H) UMAP shown in (G) indicating cellular provenance: CD133^+^ DNGR-1-traced ependymal cells in salmon and CD133^+^ non-traced cells in cyan. (I) Percentage of traced and non-traced cells in each of the clusters 0–4 from (G). Dashed line represents the proportion of CD133^+^ DNGR-1-traced cells within UMAP space (67.9%—4,723 of 6,951 cells). (J) Expression of a selected set of cluster markers from clusters 1 and 3 (from G). (K) Distribution of the top 5 Gene Ontology biological processes defined by the differentially expressed genes in each cluster. Scale bars, 500 μm (C) and 10 μm (A).

Video S7. Confocal microscopy video of uDISCO clarified spinal cord from *Clec9a*^*Cre*^*Rosa*^*LSLtdTomato*^ mouse after spinal cord contusion, related to Figure 7 Location of DNGR-1-traced cells (bright red) in response to spinal cord injury around the lesion epicentre. DNGR-1-traced cells show displacement for their central canal location toward the injury site, which was applied dorsally. Nuclei were counterstained with Hoechst (blue).

To exclude the possibility of injury-driven upregulation of DNGR-1 expression in reactive astrocytes or other astrocytic progenitors, we repeated the ventricular injury experiments in *Clec9a*^*CreERT2*^*Rosa^LSLtdTomato^* mice injected with tamoxifen immediately prior to injury (1–2 h) and during the initial recovery period (days 3 and 7 post injury). As a positive control for induction of DNGR-1 lineage tracing, DNGR-1-traced cDCs could be readily observed in the choroid plexi of injured animals (Figures 1A and S6A). In contrast, no DNGR-1-traced astrocytes were observed surrounding the injury scar or along the ependymal cell layer (Figure S6A). *Clec9a* single-molecule fluorescence *in situ* hybridization (smFISH) further confirmed that spinal cord injury does not induce DNGR-1 expression (Figure S6B). Additionally, in animals given EdU and subjected to spinal cord contusion, all DNGR-1-traced astrocytes were EdU^+^ (Figure S6C). Collectively, these data indicate that DNGR-1-traced astrocytes emerge from differentiation of the proliferative DNGR-1-traced ependymal compartment (Figure 6B). We conclude that DNGR-1-traced ependymal cells react to local CNS injury by increased proliferation and astrocytic differentiation.

To understand which signaling pathways may play a role in determining the homeostatic quiescence of DNGR-1-traced ependymal cells versus their mobilization in response to injury, we analyzed our single-cell dataset for expression of genes known to govern cell-fate decisions. Notch signaling has been shown to regulate quiescence of professional NSCs and the *Notch2* transcript could be detected in multiples clusters (Figure S6D). Additionally, the Notch target gene *Hes1*, which inhibits differentiation (Ishibashi et al., 1995), was enriched in cluster 0 (Figures 4E and S6D). Consistent with the notion that the scRNA clusters reflect dynamic ependymal cell states rather than cellular subtypes, immunostaining of uninjured spinal cord revealed that all DNGR-1-traced ependymal cells expressed HES1 protein, albeit at different levels (Figure S6E.) In contrast, HES1 expression was much more heterogeneous in spinal cords from injured animals. DNGR-1-traced cells that moved away from the central canal and acquired GFAP expressed significantly lower HES1 levels than GFAP^−^ DNGR-1-traced cells that remained in the ependymal canal (Figure 6E). Thus, DNGR-1-traced ependymal cell mobilization induced by tissue damage is associated with loss of HES1, consistent with release from a quiescent state and induction of differentiation.

### DNGR-1-traced ependymal cells constitute a distinct ependymal subset wherein resides latent stem cell potential

Although DNGR-1 lineage tracing faithfully marked ependymal cells, it did not label the entirety of the ependymal compartment. This could be due to incomplete penetrance of the Cre-mediated recombination event, resulting from a variable fraction of embryonic cells that transiently express DNGR-1 remaining unmarked. We bred DNGR-1-lineage-traced animals with the *Cle9a*^*Cre*^ and *R26*^*LSLtdTomato*^ alleles in heterozygous or homozygous configuration and confirmed that the latter displayed increased ependymal cell labeling (Figure S7A). However, double-homozygous DNGR-1-lineage-tracing mice (*Clec9a*^*Cre/Cre*^*Rosa*^*LSLtdTomato/LSLtdTomato*^) with the maximum achievable penetrance still only displayed, on average, labeling of 60% of spinal cord ependymal cells as assessed by histology (Figure S7A). To test the possibility that traced and untraced cells constitute distinct ependymal cell subtypes, we used CD133 as a pan-ependymal cell marker (Alfaro-Cervello et al., 2012; Coskun et al., 2008; Meletis et al., 2008). We first confirmed that CD133 labels all DNGR-1-traced cells in the ependymal layer, as expected (Figures 7A and S7B). We then sorted CD133^+^ DNGR-1-traced cells and CD133^+^ non-DNGR-1-traced cells from spinal cords of *Clec9a*^*Cre/Cre*^*Rosa*^*LSLtdTomato/LSLtdTomato*^ animals (Figure 7B) and tested them in the neurosphere assay (Figure 7C). Strikingly, 100% of the wells seeded with CD133^+^ DNGR-1-traced cells developed neurospheres, which expressed tdTomato and were present at an average of 10 neurospheres per well (Figure 7C). In contrast, only 7.5% of the wells seeded with CD133^+^ non-DNGR-1-traced cells developed neurospheres, which were tdTomato-negative and present on average as a single neurosphere per well (Figure 7C). Thus, the neurosphere-forming potential of CD133^+^ cells in the spinal cord is largely confined to the DNGR-1-traced compartment.

We then compared CD133^+^ DNGR-1-traced cells with CD133^+^ non-DNGR-1-traced cells by scRNA-seq (Figure 7D). Integration and embedding of 25,411 CD133^+^ non-DNGR-1-traced cells and 4,825 CD133^+^ DNGR-1-traced cells within the UMAP space resulted in unsupervised partitioning of the total 30,236 cells into 3 groups (Figure 7D) with distinct expression signatures (Figure 7E). Surprisingly, these did not all represent ependymal cells. Indeed, only cells in group 2 displayed a canonical ependymal cell signature defined by genes such as *Ccdc153*, *Fam183b*, *Tmem212*, or *Mt3* (MacDonald et al., 2021; Shah et al., 2018; Zeisel et al., 2018; Figures 7E and S7C). CD133^+^ cells in groups 1 and 3 were defined by high expression of endothelial (*Cldn5*, *Flt1*, *Pecam1*, and *Ly6c1*) or pericyte (*Pdgfrb*, *Kcnj8*, *Myl9*, and *Vtn*) genes, respectively (Figures 7E and S7C). Notably, 97.9% (4,723 of 4,825) of DNGR-1-traced cells belonged to the ependymal cell group (group 2) (Figure 7F), representing 67.9% of the total number of cells in that group (4,723 of 6,951 cells), which matches the labeling ratio observed in histological sections (Figure S7A).

To determine whether DNGR-1-tracing marked a distinct ependymal cell population, we focused on the 98.2% of cells in group 2 that expressed ependymal cell signature genes and compared the DNGR-1-traced with the non-traced fractions. This revealed that 4,946 genes were significantly differentially expressed between the two populations (Figure S7D). Although both populations expressed canonical ependymal cell genes (Figure S7E), the non-DNGR-1-traced fraction displayed an additional enrichment for genes associated with vascular/angiogenic processes (Figures S7F and S7G). GSEA of all expressed genes in each population further highlighted the hybrid angiogenic/ependymal nature of the non-traced group 2 cells. Conversely, DNGR-1-traced ependymal cells were significantly enriched in genes involved in mitotic processes and bearing binding motifs for transcription factors involved in cell cycle, such as E2F, CHAMP1, or EVI1 (Figures S7G). Thus, DNGR-1-traced ependymal cells are distinct from their non-traced counterparts and display a mitotic signature.

To understand whether the above differences were reflective of ependymal cell heterogeneity, we reclustered cells in group 2 in a new UMAP (Figures 7G and S7H). This revealed 5 different clusters, with 3 closely apposed clusters (0, 3, and 4) sitting apart from clusters 1 and 2 (Figure 7G). Mapping cellular provenance onto the UMAP revealed that cluster 1 contained exclusively non-traced cells (Figure 7H). Conversely, the DNGR-1-traced cells were overrepresented in clusters 0, 3, and 4 and underrepresented in 2 (Figures 7H and 7I), further highlighting that DNGR-1-traced ependymal cells are not identical to non-traced ependymal cells (if DNGR-1-traced ependymal cells were identical to their non-traced counterparts, they would be expected to contribute equally to each cluster, in strict proportion [67.9%; dotted line in Figure 7I] to the number of cells that they contribute to the total of the cells in group 2 of the integrated UMAPs). Gene Ontology biological process categorization, revealed that cluster 1 (containing exclusively non-DNGR-1-traced cells) displayed an angiogenic/ependymal cell signature, consistent with its aforementioned hybrid cell phenotype (Figure 7K). Conversely, cluster 3, which contained an overrepresentation of DNGR-1-traced cells, displayed a cell division signature (Figures 7J, 7K, and S7D), marked by unique expression of the canonical cell cycle genes *Mki67*, *Top2a*, and *Pbk*. Taken together, these results suggest that the partial DNGR-1 tracing of CD133^+^ ependymal cells is not fully attributable to incomplete penetrance of the recombination event that initiates lineage tracing. Rather, they support the notion that the DNGR-1-traced cells represent a distinct ependymal cell population, wherein resides latent stem cell activity.

## Discussion

Stem cell activity in ependymal cells has remained a contentious topic. Pioneering lineage-tracing studies using a human FoxJ1 promoter element first suggested the stem cell potential of ependymal cells (Barnabé-Heider et al., 2010; Carlén et al., 2009; Llorens-Bobadilla et al., 2020; Meletis et al., 2008; Sabelström et al., 2013). However, FoxJ1 expression is not confined to ependymal cells, (Beckervordersandforth et al., 2010; Benner et al., 2013; Devaraju et al., 2013; Jacquet et al., 2009) and a different FoxJ1 driver failed to reveal ependymal cell stemness (Muthusamy et al., 2018; Ren et al., 2017), perhaps because of FoxJ1 haploinsufficiency (Li et al., 2018). Here, we use a completely different driver, DNGR-1, previously studied in the context of cDC lineage tracing. DNGR-1 fate mapping showed high fidelity in ependymal cell marking, highlighted by our scRNA-seq of spinal cord DNGR-1-traced cells, which showed a 97% identity match to ependymal cells in the CNS reference atlas (Zeisel et al., 2018). Also, in the brain, DNGR-1 lineage tracing did not mark other progenitor populations, including homeostatic NSC pools in the SVZ or DG, as evidenced by the absence of traced OB interneurons or hippocampal granule neurons, respectively. Thus, the faithful ependymal cell labeling offered by the DNGR-1-lineage-tracing approach and the ability of the labeled cells to give rise to neurons, oligodendrocytes, and astrocytes *in vitro* confirms the presence of latent stem cell activity in the ependymal cell layer and corroborates the conclusions drawn by the studies using a human FoxJ1 promoter element.

Despite its accurate marking of the ependymal compartment, DNGR-1 lineage tracing did not label all ependymal cells. Fractional ependymal cell labeling could only be partly explained by incomplete penetrance of the recombination event that initiates lineage tracing. Indeed, comparison between DNGR-1-traced and non-traced cells revealed previously unrecognized ependymal cell heterogeneity. A major component of this heterogeneity was the expression of angiogenic factor-encoding genes by non-traced cells, which may correspond to the ependymal cells with a vascular signature identified previously (Luo et al., 2015). This vascular signature did not occur alongside stem cell or cell cycle genes, which were expressed predominantly in an ependymal cell cluster composed mostly of DNGR-1-traced cells and that likely corresponds to the mitotic fraction of the ependymal cell layer. It is tempting to speculate that this mitotic fraction is responsible for the self-renewal of the DNGR-1-labeled ependymal cell sub-population and is mobilized upon injury or *ex vivo* neurosphere culture. Thus, only a fraction of ependymal cells possesses latent stem cell potential and that fraction is contained within the DNGR-1-traced subset. Differential experimental capture of the DNGR-1-traced compartment may explain why some (Barnabé-Heider et al., 2010; Carlén et al., 2009; Llorens-Bobadilla et al., 2020; Meletis et al., 2008; Sabelström et al., 2013), but not other (Muthusamy et al., 2018; Ren et al., 2017; Shah et al., 2018), studies have found stem cell potential in ependymal cells. Interestingly, Troy (encoded by the *Tnfrsf19* gene) has recently been shown to mark a sub-population of ependymal cells (Stenudd et al., 2022). However, in our dataset, Troy was expressed by all ependymal cells (Figures S7I and S7J).

Adult NSCs and ependymal cells share a common lineage, both emerging from radial glia (Aimone et al., 2014; Fuentealba et al., 2015; Ortiz-Álvarez et al., 2019). Radial glia to ependymal cell commitment has been reported to occur at E15.5, when the FoxJ1-dependent ciliated program is initiated (Barnabé-Heider et al., 2010; Li et al., 2018; Meletis et al., 2008; Ortiz-Álvarez et al., 2019). However, radial glia cells are highly heterogeneous and have been reported to display fate specification for glial lineages as early as E9.5 (Malatesta et al., 2000, 2003; McCarthy et al., 2001). We found DNGR-1 expression at E11.5 in a rare population of ventricular progenitor cells, which exclusively gave rise to the traced ependymal compartment seen in adulthood. Thus, DNGR-1 expression occurs in a subset of embryonic progenitors that has exited a neurogenesis and gliogenesis trajectory but retains latent stem cell potential throughout life. While this rare DNGR-1-expressing population displayed concomitant expression of SOX2, no staining by the neuroepithelial marker nestin was observed, and its lineage relationship to the heterogeneous radial glia pool remains to be fully dissected. Furthermore, the kinetics of expansion that must occur between the emergence of the embryonic DNGR-1-expressing subset and it populating the entire CSF-lining compartment remain to be determined. Finally, which elements of the *Clec9a* enhancer and promoter regulate DNGR-1 expression in ependymal cell-committed pool versus cDC progenitors and cDC1 is presently unknown and an area for future study.

The signals that dictate homeostatic quiescence of DNGR-1-traced ependymal cells versus injury-associated differentiation also remain to be determined. The oscillatory nature of Notch signaling regulates both active and quiescent states in somatic stem cells (Harris and Guillemot, 2019; Imayoshi et al., 2013; Sueda and Kageyama, 2020). Interestingly, ependymal cells have been reported as uniquely able to survive sustained Notch signaling (Baek et al., 2006; Ishibashi et al., 1994). We found global HES1 expression in DNGR-1-traced ependymal cells during homeostasis, in accordance with their quiescent state and lack of neurogenesis or gliogenesis. Notch signaling often occurs through lateral inhibition by cell-cell contact (Sueda and Kageyama, 2020), and we hypothesize that contact loss following injury could constitute an important determinant of HES1 downregulation associated with DNGR-1-traced ependymal cell differentiation. We did not observe differentiation of DNGR-1-traced ependymal cells in old animals (our unpublished observations), suggesting that the threshold of attrition required to activate ependymal cells is superior to that resulting from aging.

Current strategies to promote CNS repair focus on stem cell transplants or direct glial reprogramming *in situ* (Barker et al., 2018). Our work identifies an embryonically derived mammalian ependymal cell subset across the entire CNS endowed with latent stem cell potential. In contrast to mammals, anamniotes such as teleost fish species display exceptional spinal cord regeneration. This is orchestrated in part by cells inhabiting the ependymal cell niche termed ependymo-radial glial cells (ERGs), which, similarly to the DNGR-1-traced ependymal cell subset described here, proliferate in response to injury and migrate toward the lesion site. Despite these similarities, mammalian rehabilitation has a poor prognosis, with long-term chronic functional impairment. Although ERGs react to injury by increasing neurogenesis, differentiation of the DNGR-1-traced ependymal subset after injury *in vivo* was restricted to the astrocytic lineage, which in turn contributed to the glial scar. This is despite the fact that DNGR-1-traced ependymal cells show neuronal differentiation potential *in vitro* and is consistent with reports of a block in mammalian neurogenesis following injury (Barnabé-Heider et al., 2010; Becker et al., 2018; Meletis et al., 2008; Stenudd et al., 2015). Given that in anamniotes, ependymal to neuronal differentiation correlates with functional motor recovery after spinal cord injury (Becker and Becker, 2015; Becker et al., 2018; Cigliola et al., 2020), coaxing the ependymal damage-responsive stem cells described here to differentiate into neurons *in vivo*, perhaps through manipulation of signals within the injury microenvironment, could represent a therapeutic strategy for the treatment of CNS injury in mammals.

### Limitations of the study

Our results argue for the presence of an embryonically fated sub-population of ependymal cells present throughout the CNS and with potential to differentiate into neurons, oligodendrocytes, or astrocytes. However, whether this means that those cells can be considered latent stem cells remains to be fully established. The tri-potency of DNGR-1-traced ependymal cells could only be revealed *in vitro*, and it will be important to assess why and how differentiation *in vivo* is restricted to astrocytes. Also, although they behave broadly similarly, we have not formally assessed possible differences in response to injury among the cells present in brain versus different parts of the spinal cord. Finally, our data are exclusively from mouse CNS. Whether analogous cells are present in other tissues and in humans remains to be investigated.

## STAR★Methods

### Key resources table

**Table:** 

REAGENT or RESOURCE	SOURCE	IDENTIFIER
**Antibodies**		

Rabbit anti-mouse/rat/human IBA-1 (polyclonal)	FUJIFILM Wako Shibayagi	Cat# 019-19741; RRID: AB_839504
AF647 anti-mouse I-A/I-E (M5/114.15.2)	BioLegend	Cat# 107618; RRID: AB_493525
AF488 anti-mouse I-A/I-E (M5/114.15.2)	BioLegend	Cat# 107616, RRID:AB_493523
eF450 anti-mouse I-A/I-E (M5/114.15.2)	Thermo Fisher Scientific	Cat# 48-5321-82, RRID:AB_1272204
Rabbit anti-mouse CD64 (#008)	Sino Biological	Cat# 50086-R008, RRID:AB_2860481
FITC anti-mouse CD64 (X54-5/7.1)	BioLegend	Cat# 139316, RRID:AB_2566556
AF488 anti-mouse CD45 (30-F11)	BioLegend	Cat# 103122, RRID:AB_493531
AF647 anti-mouse CD45 (30-F11)	BioLegend	Cat# 103124, RRID:AB_493533
Rat anti-mouse CD11b (5C6)	Bio-Rad	Cat# MCA711, RRID:AB_321292
Rabbit anti-mouse/rat/human SOX-9 (polyclonal)	Merck Millipore	Cat# AB5535, RRID:AB_2239761
Rabbit anti-mouse/rat/human Vimentin (EPR3776)	Abcam	Cat# ab92547, RRID:AB_10562134
Mouse anti-mouse/human FOXJ1 (2A5)	eBioscience, Thermo Fisher Scientific	Cat# 14-9965-82, RRID:AB_1548835
Chicken anti-GFAP (polyclonal)	Abcam	Cat# ab4674, RRID:AB_304558
Mouse anti-NeuN (A60)	Merck Millipore	Cat# MAB377, RRID:AB_2298772
Rabbit anti-mouse Polycystin-L (polyclonal)	Merck Millipore	Cat# AB9084, RRID:AB_571091
Rabbit anti-NG2 (polyclonal)	Merck Millipore	Cat# AB5320, RRID:AB_1121367
Goat anti-human Olig2 (polyclonal)	R and D Systems	Cat# AF2418, RRID:AB_2157554
Rabbit anti-mouse/human/rat PDGFr beta (Y92)	Abcam	Cat# ab32570, RRID:AB_777165
Rabbit anti-mouse HES1 (polyclonal)	Ryoichiro Kageyama, Institute for Frontier Life and Medical Sciences	N/A
AF488 anti-mouse SOX2 (Btjce)	Thermo Fisher Scientific	Cat# 53-9811-82, RRID:AB_2574479
Mouse anti-mouse/human/rat Nestin (25/NESTIN)	BD Biosciences	Cat# 611659, RRID:AB_399177
Mouse anti-oligodendrocyte marker O4 (O4)	R and D systems	Cat# MAB1326, RRID:AB_357617
Mouse anti-mouse/human/rat tubulin beta 3 (TUJ1)	BioLegend	Cat# 801201, RRID:AB_2313773
Rabbit anti-MAP2 (polyclonal)	Cell Signaling Technology	Cat# 4542, RRID:AB_10693782
Rabbit anti-mouse/human/rat GABA(B)R2 (polyclonal)	Cell Signaling Technology	Cat# 3839, RRID:AB_2232133
Mouse anti-GluN2B/NR2B (N59/36)	UC Davis/NIH	Cat# N59/36R, RRID:AB_275080
Rabbit anti-mouse/human/rat Tyrosine Hydroxylase (E2L6M)	Cell Signaling Technology	Cat# 58844, RRID:AB_2744555
Donkey anti-Rabbit IgG (H+L) AF488 conjugated	Thermo Fisher Scientific	Cat# A-21206, RRID:AB_2535792
Donkey anti-Mouse IgG (H+L) AF488 conjugated	Thermo Fisher Scientific	Cat# A-21202, RRID:AB_141607
Donkey anti-Goat IgG (H+L) AF647 conjugated	Thermo Fisher Scientific	Cat# A-21447, RRID:AB_141844
Goat anti-Rat IgG (H+L) AF647 conjugated	Thermo Fisher Scientific	Cat# A-21247, RRID:AB_141778
Goat anti-Rabbit IgG (H+L) AF647 conjugated	Thermo Fisher Scientific	Cat# A-21245, RRID:AB_2535813
Goat anti-Chicken IgY (H+L) AF488 conjugated	Abcam	Cat# ab150173, RRID:AB_2827653
Goat anti-Rabbit IgG (H+L) AF488 conjugated	Thermo Fisher Scientific	Cat# A-11034, RRID:AB_2576217
Goat anti-Mouse IgG (H+L) AF488 conjugated	Thermo Fisher Scientific	Cat# A-11029, RRID:AB_2534088
Donkey anti-rabbit IgG (H+L) AF555 conjugated	Thermo Fisher Scientific	Cat# A-31572, RRID:AB_162543
AF488 anti-mouse CD133 (Prominin-1) (13A4)	Thermo Fisher Scientific	Cat# 53-1331-80, RRID:AB_529615
Rat anti-mouse CD16/32 (2.4G2)	BD Biosciences	Cat# 553142, RRID:AB_394657
PE/Cyanine7 anti-mouse CD45.2 antibody (104)	BioLegend	Cat# 109829, RRID:AB_1186103
PerCP/Cy5.5 anti-mouse CD11c, (N418)	BD Biosciences	Cat# 560584, RRID:AB_1727422
FITC anti-mouse CD103 (M290)	BD Biosciences	Cat# 557494, RRID:AB_396731
APC-eF780 anti-mouse CD11b (M1/70)	eBioscience	Cat# 47-0112-82, RRID:AB_1603193
AF647 anti-mouse XCR1 (ZET)	BioLegend	Cat# 148214, RRID:AB_2564369
Rabbit anti-RFP (polyclonal)	Rockland	Cat# 600-401-379, RRID:AB_2209751
Anti-mouse CD45 MicroBeads	Miltenyi Biotec	Cat# 130-052-301, RRID:AB_2877061

**Chemicals, peptides, and recombinant proteins**		

Tamoxifen	Sigma-Aldrich	Cat# T5648
(Z)-4-Hydroxytamoxifen	Sigma-Aldrich	Cat# H7904
Peanut oil	Sigma-Aldrich	Cat# P2144
EdU (5-ethynyl-2’-deoxyuridine)	Thermo Fisher Scientific	Cat# E10187
*Tert*-Butanol	Sigma-Aldrich	Cat# 360538
Dichloromethane	Sigma-Aldrich	Cat# 270997
Benzyl alcohol	Sigma-Aldrich	Cat# 305197
Benzyl benzoate	Sigma-Aldrich	Cat# B6630
Diphenyl ether	Alfa Aesar	Cat# A15791
DL-alpha-Tocopherol	Alfa Aesar	Cat# A17039
Laminin	Sigma-Aldrich	Cat# L2020
Heparin	Sigma-Aldrich	Cat# H3393
T3 hormone (3,3′,5-Triiodo-L-thyronine sodium salt)	Sigma-Aldrich	Cat# T2752
Animal-Free Recombinant Murine FGF basic	Peprotech	Cat# AF-450-33
Animal-Free Recombinant Murine EGF	Peprotech	Cat# AF-315-09
Recombinant Murine IGF-I	Peprotech	Cat# 250-19
Recombinant Murine BMP-4	Peprotech	Cat# 315-27

**Critical commercial assays**		

Click-iT™ EdU Cell Proliferation Kit for Imaging, Alexa Fluor™ 647 dye	Thermo Fisher Scientific	Cat# C10340
RNAscope Multiplex FL V2	Biotechne	Cat# 323110
TSA plus Cyanine 5	Perkin Elmer	Cat# NEL745001
Neural Dissociation Kit (P)	Miltenyi biotec	Cat# 130-092-628
Myelin removal beads II	Miltenyi biotec	Cat# 130-096-733, SCR_020279
QIAShredder	QIAGEN	Cat# 79656
RNeasy Mini kit	QIAGEN	Cat# 74004

**Deposited data**		

Mouse Brain Atlas	(Zeisel et al., 2018)	SRP135960
Bulk RNA sequencing of DNGR-1 traced or hippocampal neural stem cells and astrocytic progenies	This paper	GSE145824
Single Cell RNA sequencing of spinal cord DNGR-1 traced ependymal cells	This paper	GSE146226
Single Cell RNA sequencing of spinal cord CD133^+^ DNGR-1 traced and CD133^+^ non-traced cells	This paper	GSE202959

**Experimental models: Organisms/strains**		

*Clec9a*^*Cre*^*Rosa*^*LSLtdTomato*^	(Schraml et al., 2013)	N/A
*Clec9a*^*Cre*^*Rosa*^*LSLEYFP*^	(Schraml et al., 2013)	N/A
*Clec9a*^*Cre*^*Rosa*^*LSLtdTomato*^*Batf3*^*-/-*^	This paper	N/A
*Clec9a*^*Cre*^*Rosa*^*LSLtdTomato*^*Flt3l*^*-/-*^	This paper	N/A
*Clec9a*^*tdTomato*^	This paper	N/A
*Clec9a*^*CreERT2*^*Rosa*^*LSLtdTomato*^	This paper	N/A

**Oligonucleotides**		

RNAscope 2.5 LS Probe -Mm-Clec9a-01	Biotechne	Cat# 537738

**Software and algorithms**		

Imaris (v.9.1.2)	Imaris software	https://imaris.oxinst.com/
FlowJo (v10.8.1)	FlowJo	https://www.flowjo.com/
ZEN black	ZEISS	https://www.zeiss.com/microscopy/int/products/microscope-software/zen.html
Prism (GraPad 9.2.0)	GraphPad software	https://www.graphpad.com/scientific-software/prism/
Seurat R package (v3)	Seurat	https://satijalab.org/seurat/
Adobe InDesign (v17.1)	Adobe System	https://www.adobe.com/
Biorender	Biorender	https://biorender.com/
FIJI (v2.1.0/1.53c)	ImageJ	https://imagej.nih.gov/ij/
R studio (3.5)	R software	https://www.rstudio.com/

**Other**		

Collagenase IV	Worthington	Cat# LS004188
DNAse I	Roche	Cat# 11284932001
LIVE/DEAD Fixable Blue Dead Cell Stain Kit	Thermo Fisher Scientific	Cat# L34962
Fixation Medium A	Nordic MUbio	Cat# GAS-002A-1
StemPro™ Accutase™ Cell Dissociation Reagent	Thermo Fisher Scientific	Cat# A1110501
N-2 MAX Media Supplement	R and D Systems	Cat# AR009
NeuroCult™ Proliferation Supplement	Stem Cell Technologies	Cat# 05701
Matrigel	Corning	Cat# 354230
B-27™ Supplement (50X), serum free	Thermo Fisher Scientific	Cat# 17504044
Vybrant™ DiO Cell-Labeling Solution	Thermo Fisher Scientific	Cat# V22886
FluoroMyelin™ Green Fluorescent Myelin Stain	Thermo Fisher Scientific	Cat# F34651

### Resource availability

#### Lead contact

Further information and requests for resources should be directed to and will be fulfilled by the lead contact, Caetano Reis e Sousa (caetano@crick.ac.uk).

#### Material availability

All mouse lines generated in this study are available from the lead contact.

### Experimental model and subject details

#### Mice

*Clec9a*^*CreERT2*^ (carrying allele *Clec9a^tm4.1Crs^*) mice were generated by conventional gene targeting (knock-in) adding P2A CreERT2 to the Clec9a gene. *Clec9a*^*tdTomato*^ (carrying allele *Clec9a^tm5.1Crs^*) mice were generated by knock-in of tdTomato with a BGH_pA at the ATG in exon 1 of the Clec9a gene. An FRT-flanked Neo cassette in the original constructs of both strains was removed by crossing to an FLPe expressing strain. Generation of *Clec9a*^*CreERT2*^ and *Clec9a*^*tdTomato*^ mice was contracted commercially (Ozgene, Perth, WA, Australia). Allele names refer to The Francis Crick Institute Nomenclature. *Clec9a*^*Cre*^ (Schraml et al., 2013), *Rosa*^*LSLtdTomato*^, *Rosa*^*LSLEYFP*^ (Madisen et al., 2010), *Batf3*^*-/-*^ (Hildner et al., 2008) and *Flt3l*^*-/-*^ (McKenna et al., 2000) were described previously. All mice were bred at The Francis Crick Institute under specific pathogen-free conditions. All genetically modified mouse lines were backcrossed to C57BL/6j and six to fourteen-week-old mice were used in all experiments unless otherwise specified. Mouse genotypes from ear biopsies were determined using real time PCR with specific probes designed for each gene (Transnetyx, Cordova, TB). All animal experiments were performed in accordance with national and institutional guidelines for animal care and were approved by the Francis Crick Institute Biological Resources Facility Strategic Oversight Committee (incorporating the Animal Welfare and Ethical Review Body) and by the Home Office, UK.

#### Primary cell cultures

##### Neurosphere assay, neural stem cell adherent cultures and differentiation

*Neurosphere assay*. *Clec9a*^*Cre*^*Rosa*^*LSLtdTomato*^ or *Clec9a*^*CreERT2*^*Rosa*^*LSLtdTomato*^ mice were sacrificed with i.p. injection of pentobarbital and perfused with 20ml of sterile PBS. Brains or spinal cords were digested into a single-cell suspension using Neural Tissue Dissociation kit (P) (Miltenyi) and 1.5x10^5^ cells per cm^2^ were seeded per well (96 well plates) in DMEM:F12 (Thermo Fisher Scientific) supplemented with 0.015M KCl, 1% BSA, Neurocult (Stem Cell Technologies), bFGF (10ng/ml, Peprotech), EGF (20ng/ml, Peprotech), Heparin (2ug/ml, Sigma), antibiotics and glutamine (Neurosphere medium). 10-14 days after culture, plates were inspected using an EVOS XL Core microscope (Thermo Fished Scientific) and transmitted light and fluorescence images acquired. Wells containing tdTomato^+^ neurospheres were collected and neurospheres dissociated with StemPro Accutase (Thermo Fished Scientific) to generate a single-cell suspension. TdTomato^+^ cells were FACS sorted from primary neurospheres after StemPro Accutase dissociation and DAPI staining (to exclude dead cells) using an Aria Fusion (BD) with a 100μm nozzle. Purity checks were performed on sorted cells (<95% tdTomato^+^).

To assess potential DNGR-1 up-regulation triggered by *in vitro* culture, brains or spinal cords tissue from *Clec9a*^*CreERT2*^*Rosa*^*LSLtdTomato*^ mice were processed as above and further incubated with (Z)-4OH-TAM (500nM, Sigma) or DMSO at the onset of the assay and media was half-exchanged every 2/3 days.

For comparison of the neurosphere-forming potential between CD133^+^ DNGR-1-traced and CD133^+^ non-traced cells, spinal cords from *Clec9a*^*Cre/Cre*^*Rosa*^*LSLtdTomato/LSLtdTomato*^ mice were digested and myelin-depleted as above and stained with anti-CD133 AF488 (1:100, clone 134A, BD) and anti-CD45 AF647 (1:100, clone 30F-11, Biolegend). CD133^+^CD45^-^ DNGR-1-traced or non-traced cells were FACS-sorted on an Aria Fusion (BD) with a 100μm nozzle and 100 cells from each compartment seeded per well (96 well plate) in 100ul of neurosphere medium for 14 days.

*Adherent cultures*. Adherent cultures (Conti et al., 2005; Pollard et al., 2006) were established by plating (previously sorted) neurosphere-derived tdTomato^+^ single-cell suspensions in flasks pre-coated for at least 1 hour with DMEM:F12 (Thermo Fisher Scientific) supplemented with N-2 Max supplement (R&D systems), bFGF (20ng/ml, Peprotech), EGF (20ng/ml, Peprotech), Heparin (5ug/ml, Sigma), Laminin (2ug/ml, Sigma), antibiotics and glutamine. (NSC medium).

*Astrocyte differentiation*. To induce astrocyte differentiation (Bonaguidi et al., 2005), NSCs (5x10^4^ cells) derived from adherent cultures were cultured in poly-l-lysine (20mg/ml) coated coverslips (13mm) in NSC medium overnight. On the following day, media was replaced with DMEM:F12 (Thermo Fisher Scientific) supplemented with BMP4 (20ng/ml, Peprotech) (astrocyte differentiation medium). After 3 days, media was replaced by freshly prepared astrocyte differentiation medium and cells incubated for another 3 days before cultures were terminated.

*Oligodendrocyte differentiation*. To induce oligodendrocyte differentiation (Hsieh et al., 2004; Panciera et al., 2016), NSCs (5x10^4^ cells) derived from adherent cultures were cultured in poly-l-lysine (20mg/ml) coated coverslips (13mm) in NSC medium overnight. On the following day, media was replaced with Neurobasal medium (Thermo Fisher Scientific) supplemented with IGF-I (500ng/ml, Peprotech), 1xB27 supplement (Thermo Fisher Scientific), and T3 (30ng/ml, Sigma), antibiotics and glutamine (oligodendrocyte differentiation medium). After 3 days, media was replaced by freshly prepared oligodendrocyte differentiation medium and cells incubated for another 3 days before cultures were terminated.

*Neuronal differentiation*. To induce neuronal differentiation (Choi et al., 2014; Panciera et al., 2016), NSCs (5x10^4^ cells) derived from adherent cultures were mixed with Matrigel (Corning) (diluted 1:15 in Neurobasal medium supplemented with 1x B27 + vitamin A (Thermo Fisher Scientific), antibiotics and glutamine (neuronal differentiation medium)) and added to a tissue culture well (24 wp) and allowed to form a thin-gel layer (1hr, 37C). After 3 days, media was replaced by freshly prepared neuronal differentiation medium and cells incubated for another 3 days before cultures were terminated.

##### *In vitro* cDC culture

*In vitro* cDC cultures were generated as described in Helft et al. (2015). Briefly, differentiated cDCs cells were prepared from day 9 bone marrow cultures grown from *Clec9a*^*CreERT2*^*Rosa*^*LSLtdTomato*^ mice in the presence of Flt3L (150 ng ml^−1^) exposed to (Z)-4OH-TAM (500nM, Sigma) or DMSO.

### Method details

#### Radiation bone marrow chimeras

*Clec9a*^*Cre*^*Rosa*^*LSLEYFP*^ mice were irradiated (two doses of 6.6 Gray separated by 4hours). On the following day, mice were i.v. injected with 2x10^6^ total bone marrow cells isolated from *Clec9a*^*Cre*^*Rosa*^*LSLtdTomato*^ mice. Animals were analyzed after 8 weeks or 1 year after reconstitution.

#### EdU administration in drinking water

10 week old *Clec9a*^*Cre*^*Rosa*^*LSLtdTomato*^ mice were treated with EdU (Thermo Fisher Scientific) in drinking water (0.75mg/ml EdU + 1% sucrose) for a period of 30 days. This solution was protected from light and prepared fresh and changed every 3 days.

#### Immunofluorescence and confocal microscopy

*Clec9a*^*Cre*^*Rosa*^*LSLtdTomato*^ mice were sacrificed with i.p. injection of pentobarbital and perfused with 20ml of PBS followed by 10 ml of 4% paraformaldehyde (PFA). Brains or spinal cords were dissected and further fixed in 4% PFA overnight (ON) at 4C. On the following day, tissues were washed in PBS for 1hr at 4C with agitation and transferred to 30% sucrose in PBS for 24hours at 4C. Tissues were finally embedded in O.C.T. (Tissue-Tek) and stored and -80C. Frozen tissue sections (10-20μm) were produced on a cryostat (Leica), mounted on SuperFrost Plus glass slides (Thermo Fisher Scientific) and stored at -80C. Before staining, sections were thawed at room temperature for 20 minutes, re-hydrated in PBS and blocked with 3% BSA, 0.3% Triton X-100 in PBS (blocking buffer) for 1 hour at room temperature in a humid chamber. Primary antibodies were diluted in blocking buffer and incubated ON at 4C in a humid chamber. TdTomato in direct reporter and inducible tracer was detected with anti-RFP.

For EdU incorporation detection, sections were permeabilized and incubated for 5 minutes with 3% BSA, 0.3% Triton X-100 and incubated with a Click-iT reaction cocktail solution (Click-iT Imaging kit, Thermo Fisher Scientific) containing 500μm AF647 PCA for 30 minutes at room temperature. Nuclei were counterstained with Hoechst. Images were quantified using ImageJ/FIJI for the proportion of DNGR-1-traced cells which had incorporated EdU per section in uninjured or injured spinal cords (lesion periphery and injury core).

For FoxJ1 and Hes1 staining, sections were subjected to epitope retrieval using citrate buffer (10mM NaCitrate, 0.05% Tween 20, pH 6, 3minutes at 80C) before blocking. Secondary antibodies were diluted in blocking buffer and incubated for 1 hour at room temperature in a humid chamber. Nuclei were counterstained with Hoechst and sections were mounted with ProLong Diamond Antifade (Thermo Fisher Scientific).

For the detection of DNGR-1 expressing cells in E11.5 *Clec9a*^*tdTomato*^ embryos or adult spinal cord tissue, sections were stained with a rabbit anti-RFP (rockland) polyclonal antibody followed by an AF555 donkey anti-rabbit secondary antibody.

Hes1 staining was quantified by drawing a nuclear mask defined by Hoechst staining and measuring the mean gray value intensity for Hes1 fluorescence channel on Fiji/ImageJ software (Schindelin et al., 2012).

To map the distribution of DNGR-1-traced cells across the brains of *Clec9a*^*Cre*^*Rosa*^*LSLtdTomato*^ mice, brains were dissected and fixed in 10% neutral buffered formalin (NBF) ON at room temperature and moved to 70% ethanol on the following day. Tissues were embedded in paraffin and 4μm sections cut on a microtome. Paraffin was removed from sections by serial incubation through graded xylene and ethanol solutions.

Quantification of ependymal cell labeling in *Clec9a*^*Cre*^*Rosa*^*LSLtdTomato*^ animals of different penetrance made by carrying *Clec9a*^*Cre*^ or *Rosa*^*LSLtdTomato*^ alleles in hetero- or homozygosity was performed in 20μm spinal cord sections by enumerating the fraction of DNRG-1-traced cells present in the ependymal cell layer.

For immunofluorescence of NSCs or astrocytes, oligodendrocytes or neuronal differentiated cultures, cells were cultured in poly-L-lysine coated (20mg/ml) coverslips, fixed (4%PFA, 30 minutes, room temperature), permeabilised (0.03% triton X-100, 30 minutes, room temperature) and blocked (3% BSA, 1 hour, room temperature) before being stained with primary antibodies (diluted in blocking buffer, 16 hours, 4C) and secondary antibodies (diluted in blocking buffer, 1 hour, room temperature). Coverslips were finally mounted in ProLong Diamond Antifade containing DAPI. Samples were imaged on a Zeiss LSM880 inverted confocal microscope and NSC cultures and their differentiated progeny samples were imaged using the Airyscan module.

#### Single molecule fluorescent *in situ* hybridisation (smFISH)

*Clec9a* mRNA expression was detected using the RNAscope Multiplex Fluorescent v2 assay combined with immunofluorescence (ACD) following manufacturer’s instructions. Lymph nodes or injured spinal cords from PBS and 4% paraformaldehyde (PFA) perfused *Clec9a*^*+/Cre*^*Rosa*^*LSLtdTomato*^ mice were further fixed with 4% PFA (16h, 4C) and cryoprotected with 30% sucrose (16h, 4C) prior to making 10 μm thick frozen sections. *Clec9a* mRNA was detected using probe mm-Clec9a-01 (537731, ACD) in conjunction with TSA633 (NEL745001, Perkin Elmer). Sections were further stained with a rat anti-CD45 (1:100, clone 30-F11, Biolegend), detected with a donkey anti-rat AF488 (1:400, Thermo Fischer Scientific); and with a rabbit anti-RFP (1:600, polyclonal, Rockland), detected with a donkey anti-rabbit AF555 (1:400, polyclonal, Thermo Fischer Scientific). Nuclei were counterstained with Hoechst. Images were acquired on a LSM880 inverted confocal microscope (Zeiss). Number of *Clec9a* puncta per cell were quantified using ImageJ/FIJI. Background level of the assay was determined by counting the maximum number of *Clec9a* puncta in *Clec9a*-deficient animals (*Clec9a*^*Cre/Cre*^*Rosa*^*LSLtdTomato/LSLtdTomato*^).

#### Flow cytometry

Meninges, brains or spinal cords of PBS perfused *Clec9a*^*Cre*^*Rosa*^*LSLtdTomato*^ mice were cut into small pieces and digested with Collagenase IV (200U/ml, Worthington) and DNase I (0.2mg/ml, Roche) in RPMI for 60 minutes at 37C. Digested tissues were strained though a 70μm cell strainer (BD Bioscience). Leukocytes were enriched in brain samples by Percoll gradient centrifugation (GE Healthcare). Nine parts Percoll were combined with one-part 10x PBS to obtain 100% Percoll. Cells were resuspended in 70% Percoll in PBS or HBSS/RPMI, overlaid with 37% and 30% Percoll and centrifuged at room temperature for 30min at 2000rpm without braking. Cells were collected at the 70/37% interface. DNGR-1 traced cDC1s or cDC2s were identified in meningeal and brain samples as singlets, LIVE, tdTomato+, CD45+ MHCII+, CD11c+ CD64-, CD103+ CD11b- (cDC1) or CD103- CD11b+ (cDC2). Spinal cord samples were not subjected to Percoll enrichment. All samples were Fc-blocked with anti-CD16/32 (clone 2.4G2, 1:100 BD Bioscience) prior to staining. Antibodies used: anti-CD45 PeCy7 (1:400, clone 104, Biolegend), anti-MHCII eF450 (1:200, clone M5.114.15.2, eBioscience), anti-CD11c PerCP Cy5.5 (1:100, clone N418, BD Bioscience), anti-CD64 AF647 (1:200, clone X54-5/7.1, Biolegend), anti-CD103 FITC (1:100, clone M290, BD Bioscience), anti-CD11b APCeF780 (1:200, clone M1/70, eBioscience). cDC1s were identified in *in vitro* cDC cultures by staining with anti-XCR1 AF647 (1:100, clone ZET, Biolegend). Dead cells were exclude by LIVE/DEAD Blue staining (Thermo Fisher).

#### Correlative light and electron microscopy

##### Fluorescence super-resolution microscopy

*Clec9a*^*Cre*^*Rosa*^*LSLtdTomato*^ mice were sacrificed with pentobarbital and perfused with 20ml of pPBS followed by 10 ml of 4% paraformaldehyde (PFA). Spinal cords were dissected and further fixed in 4% PFA overnight (ON) at 4C. Spinal cords were embedded in 4% low melting point agarose (Life technologies) and 100μm vibratome sections were produced. Nuclei were counterstained with Hoechst and sections were imaged on a Zeiss LSM880 inverted confocal microscope using the Airyscan super-resolution module using Z-step correction (Z-step=100nm).

Once fluorescence microscopy was completed, the vibratome slices were further fixed in 2.5% glutaraldehyde and 4% formaldehyde in 0.1 M phosphate buffer (pH 7.4) and processed according to the method of the National Centre for Microscopy and Imaging Research (Deerinck et al., 2010), before flat embedding in Durcupan resin between sheets of Aclar plastic.

##### SBF SEM data collection

Serial blockface scanning electron microscopy (SBF SEM) data was collected using a 3View2XP (Gatan, Pleasanton, CA) attached to a Sigma VP SEM (Zeiss, Cambridge). Flat embed vibratome slices were cut out and mounted on pins using conductive epoxy resin (Circuitworks CW2400). Each slice was trimmed using a glass knife to the smallest dimension in X and Y whilst retaining all the tissue, and the surface polished to reveal the tissue before sputter coating with a 2 nm layer of platinum, and loading in the 3View2XP. Two SBF SEM datasets were collected, both of which fully contained the fluorescence microscopy volume. Backscattered electron images were acquired using the 3VBSED detector at 8,192^∗^8,192 pixels with a dwell time of either 5 or 4 μs (10 nm reported pixel size for a horizontal frame width of 81.92 μm) and 50 nm slice thickness. The SEM was operated in variable pressure mode at a chamber pressure of either 10 or 5 pascals, with high current mode inactive. The 30 μm aperture was used, with an accelerating voltage of 2.5 kV. Dataset 1 comprised a total of 1,180 images, representing a depth of 59 μm, and volume of 395,942 μm^3^; dataset 2 comprised a total of 1,296 images, representing a depth of 64.8 μm, and volume of 434,865 μm^3^.

##### Image processing

Downstream image processing was carried out using Fiji/ImageJ (Schindelin et al., 2012). The images were batch converted to 8-bit tiff format, denoised using Gaussian blur (0.75 pixel radius), resharpened using two passes of unsharp mask (4 pixel radius 0.3 strength, 1 pixel radius 0.2 strength) and contrast enhanced (saturated pixels 0.2%, normalise); these parameters were tailored to suit the resolution and image characteristics of the datasets. Image registration was carried out using the ‘align virtual stack slices’ plugin, with a translation model used for feature extraction and registration. The aligned image stacks were calibrated for pixel dimensions, and cropped to regions of interest as required. To generate a composite of the volumes, Bigwarp (Bogovic et al., 2015; Russell et al., 2016) was used to map each fluorescence microscopy channel into the electron microscopy volume. After exporting the transformed fluorescence microscopy volumes from Bigwarp, the datasets were combined, and brightness/contrast adjusted for optimal on-screen presentation.

#### Multiphoton microscopy

300μm vibratome sections from spinal columns of *Clec9a*^*Cre*^*Rosa*^*LSLtdTomato*^ mice were imaged using a Zeiss 710 NLO laser scanning multiphoton microscope equipped with a 20x 1.0 NA immersion lens. A pulsed Ti:sapphire laser (Spectra Physics MaiTai HP DeepSee) tuned to 900nm was used for excitation and emission wavelengths were detected through band-pass filters of 380-485nm (second harmonic signal) and 640-690nm (tdTomato). Images were analyzed and channels adjusted using Imaris (Bitplane).

#### Optical clearing of tissues

Whole central nervous system (CNS), spinal cords or E11.5 embryos from *Clec9a*^*Cre*^*Rosa*^*LSLtdTomato*^mice were optically cleared using uDISCO (Pan et al., 2016). Briefly, mice were sacrificed with pentobarbital and perfused with 20ml of PBS followed by 10 ml of 4% paraformaldehyde (PFA). Tissues were dissected and further fixed in 4% PFA overnight (ON) at 4C. On the following day, tissues were washed in PBS for 1 hour at 4C and nuclei were stained with Hoechst diluted in PBS and incubated ON at 4C. Tissues were dehydrated by serial incubation in *tert*-butanol solutions of increasing concentration (Sigma, 360538) 30% vol *tert*-butanol, 50% vol *tert*-butanol, 70% vol *tert*-butanol, 80% vol *tert*-butanol, 90% vol *tert*-butanol and 96% vol *tert*-butanol. Spinal cord and embryos were incubated in each solution for at least 4 hours at room temperature with agitation, protected from light. Whole CNS was further incubated with 100% vol *tert*-butanol and incubated in each solution for at least 16 hours at 35C with agitation, protected from light. Whole CNS was further incubated with a delipidation solution of pure dichloromethane (Sigma, 270997) for 70 minutes at room temperature with agitation. Finally, all tissues were incubated in BABB:D4 - prepared by mixing BABB (benzyl alcohol + benzyl benzoate 1:2, Sigma, 305197 and B6630) with diphenyl ether (DPE) (Alfa Aesar, A15791) at a ratio of 4:1 and adding 0.4% vol DL-alpha-tocopherol (Vitamin E) (Alfa Aesar, A17039) for at least 48hrs with agitation before imaging.

#### Light-sheet microscopy

uDISCO clarified CNS, spinal cord or embryo from *Clec9a*^*cre*^*Rosa*^*LSLtdtomato*^ mice were imaged on a Miltenyi-LaVision BioTec Ultramicroscope II light-sheet microscope. Tissues were mounted on a sample holder and imaged in BABB-D4 solution with an Olympus MVPLAPO 2x /0.5 NA with a protective dipping cap (WD > 5.7 mm). Tissues were excited with a bi-directional 561nm wavelength distributed across three Gaussian light-sheets with a NA of 0.09 and exposed for 200ms. The step size between each image was 5 μm. Images from both light-sheets were acquired with an Andor Zyla 5.5 sCMOS camera and merged using the blend function in Imspector Pro software. A zoom of 1.25x-1.6x was used for imaging of E11.5 embryos. A zoom of 0.63x was used for imaging of whole CNS and volumes were stitched using BigStitcher (Hörl et al., 2019) in Fiji/ImageJ software (Schindelin et al., 2012). DNGR-1 traced surfaces were generated using Imaris software (Bitplane). Given the lower tdTomato fluorescence of DNGR-1 traced cDCs (Figures S1C and S5C) a low laser power was used to preferentially illuminate the bright tdTomato ependymal cell compartment in injured spinal cords (Figure 7A).

#### Embryonic tracking and in utero induction of DNGR-1 lineage tracing

Embryos were dated taking the day of the plugs as embryonic day (E) 0.5 and processed for histological analysis or wholemount clarification as detailed above.

For in utero induction of DNGR-1lineage tracing, *Clec9a*^*CreERT2*^*Rosa*^*LSLtdTomato*^ females at E10.5 and E11.5 stages of pregnancy were intraperitoneally injected with 5ul/g of body weight of a tamoxifen (T5648, Sigma) solution (20mg/ml) made in peanut oil (P2144, Sigma).

#### Spinal cord injury contusion model

Only female animals were used in spinal cord injury experiments. Two weeks prior to surgery mice went through a period of handling and acclimatization, during which body weight was assessed to ensure ideal surgical weight (18-20 g). Animals (10-11 weeks old) were anesthetized using a cocktail of ketamine (120 mg/kg) and xylazine (16 mg/kg) administered by intraperitoneal (ip) injection. All surgeries were performed under aseptic conditions. For spinal contusion injuries, a laminectomy of the ninth thoracic vertebra (T9), identified based on anatomical landmarks, was first performed (Harrison et al., 2013), followed by a moderate (75 kdynes; displacement: 600-700 μm) contusion using the Infinite Horizon Impactor (Precision Systems and Instrumentation, LLC.) (Scheff et al., 2003). After spinal cord injury, the muscle and skin were closed with 4.0 polyglycolic absorbable sutures (Safil, G1048213). Animals were injected with 0.5 ml of saline subcutaneously (s.c.) then placed onto warmed cages (35°C) until they recovered from anaesthesia and for the following recovery period (3 days). To prevent dehydration, mice were supplemented daily with saline (0.5 ml, s.c.) for the first 5 dpi. Bladders were manually voided twice daily for the duration of the experiment (Basso et al., 2006).

##### White matter sparing analysis to determine lesion epicentre

Segments from injured spinal cords containing the T9 region were processed for immunofluorescence as detailed above. 10μm-thick frozen serial sections spaced 100μm apart were stained with Fluoromyelin Green (Thermo Fisher Scientific) to identify white matter regions (myelinated) and counterstained with Hoechst. Sections were imaged on a Zeiss LSM880 inverted confocal microscope and images analysed with Fiji/ImageJ. Total cross-sectional area (TCA) and white matter area (WMA, fluoromyelin^+^) were calculated using Fiji/ImageJ and the proportional relationship WMA/TCA was calculated across the entire series of sections. The lowest WMA/TCA value (corresponding to the section with the smallest myelinated area) was defined as the lesion epicentre and lesion coordinates (rostrally and caudally) were extrapolated.

#### EdU administration to spinal cord injured animals

Mice were daily I.P. injected with 50mg/Kg EdU in 200ul of PBS for 7 consecutive days after contusion spinal cord injury.

#### Ventricular injury

Mice were anesthetized with 2% isofluorane, the head shaved and placed in a custom-built stereotaxic apparatus where anesthesia was maintained throughout the entire experiment with 0.5–1% isoflurane in O_2_. A craniotomy was performed bilaterally (0.5mm post-bregma, 1.5mm and -1.5mm mediolateral) and the dura was removed around the penetration site. A 30g needle (311.2μm outer diameter) was mounted on the stereotaxic apparatus, dipped into the green lipophilic dye DiOC_18_(3) (Thermo Fisher Scientific) and a 2.5mm dorsoventral incision was made bilaterally. Skin was sutured and mice allowed to recover in a heating chamber (35C) until fully recovered from anaesthesia. *Clec9a*^*CreERT2*^*Rosa*^*LSLtdTomato*^ mice were intraperitoneally injected with 5ul/g of body weight of a tamoxifen (T5648, Sigma) solution (20mg/ml) made in peanut oil (P2144, Sigma) 1-2 hours before injury and after 3- and 7-days post injury. 4 weeks after ventricular injury, mice were sacrificed as above and brains dissected. Serial 100μm vibratome sections were cut and stained as in described elsewhere (Cabeza-Cabrerizo et al., 2019).

#### Bulk RNA sequencing of NSC and astrocyte cultures

##### Sample preparation

Samples were prepared as illustrated in Figure S4D. Briefly, passage 10 NSCs adherent cultures generated from spinal cords of *Clec9a*^*cre*^*Rosa*^*LSLtdtomato*^, *Clec9a*^*cre*^*Rosa*^*LSLEYFP*^ or from the hippocampi of *Glast*^*Cre*^*Huwe1*^*fl*^*Rosa*^*LSLEYFP*^mice were either passaged, re-seeded as NSCs in adherent conditions or induced to differentiate into astrocytes (see above). NSCs seeded in adherent conditions were recovered with StemPro accutase (Thermo Fisher Scientific) after 4 days, whilst differentiated astrocytes were recovered after 6 days. Cells from either condition were lysed with RLT buffer (QIAGEN) supplemented with 2-mercapoethanol and cell lysates stored at -80C. Three replicates for each cell fate (3x10^6^NSCs or astrocytes) generated from passages P10, P11 and P12 adherent NSCs were sequenced.

##### Library preparation and RNA-sequencing

Biological replicate libraries were prepared using the polyA KAPA mRNA HyperPrep Kit and sequenced on Illumina HiSeq 4000 platform, generating ∼26 million 75bp single-end reads per sample. The RSEM package (version 1.3.30) (Li and Dewey, 2011) in conjunction with the STAR alignment algorithm (version 2.5.2a) (Dobin et al., 2013) was used for the mapping and subsequent gene-level counting of the sequenced reads with respect to Ensembl mouse GRCm.38.89 version transcriptome. Normalisation of raw count data and differential expression analysis was performed with the DESeq2 package (version 1.18.1) (Love et al., 2014) within the R programming environment (version 3.4.3). Differentially expressed genes were defined as those showing statistically significant differences between the Stem Cell and Astrocytes Groups (FDR <0.05). Gene lists ranked by the Wald statistic were used to look for pathway and biological process enrichment using the Broad’s GSEA software (version 2.1.0) with genesets from MSigDB (version 6) (Subramanian et al., 2005).

#### Single-cell RNA sequencing

##### Single-cell sample preparation for spinal cord DNGR-1 traced cells:

*Clec9a*^C*re*^*Rosa*^*LSLtdTomato*^ mice were sacrificed with i.p. injection of pentobarbital, perfused with 20ml of sterile PBS, spinal cords dissected and digested into a single-cell suspension using Neural Tissue Dissociation kit (P) (Miltenyi Biotec) following manufacture instructions. Sample was enriched for ependymal cells by further processing with myelin removal magnetic beads II (Miltenyi Biotec) to remove myelinated cells by passage through a MACS column (Miltenyi Biotec). Cells were then stained with FITC-conjugated anti-CD45.2 (clone 104), incubated with anti-FITC magnetic MicroBeads (Miltenyi Biotec) and put through another MACS column to remove CD45^+^ cells. Cells were finally stained with APC-conjugated CD45 (clone 30-F11) and DAPI (to exclude dead cells) and single DNGR-1 traced (tdTomato^+^) CD45^-^ DAPI^-^ cells were FACS sorted on an Aria Fusion (BD) with a 100μm nozzle. QC confirmed viability <95% and cells were immediately loaded onto 10X Genomics Chromium according to manufacture instructions.

##### Single-cell sample preparation for comparison between CD133+ DNGR-1 traced and CD133+ non-traced cells from spinal cords

Spinal cords from *Clec9a*^*Cre/Cre*^*Rosa*^*LSLtdTomato/LSLtdTomato*^ mice were processed as above. Myelin-depleted single cell suspensions were stained with APC-conjugated CD45 (clone 30-F11) and AF488-conjugated CD133 (clone 13A4) and DAPI (to exclude dead cells). Single DAPI^-^ CD45^-^ CD133^+^ DNGR-1 traced or DAPI^-^ CD45^-^ CD133^+^ non- traced cells were FACS sorted on an Aria Fusion (BD) with a 100μm nozzle into two different Eppendorf tubes. QC confirmed viability <95% and cells were immediately loaded onto 10X Genomics Chromium according to manufacture instructions.

##### Single-cell library preparation and RNA-sequencing

Library generation for 10x Genomics analysis were performed following the Chromium Single Cell 3′ Reagents Kits (10x Genomics) and sequenced on an Hiseq4000 (Illumina), to achieve an average of ∼280,000 reads per cell. Raw reads were initially processed by the Cell Ranger v.3.0.2 pipeline, which deconvolved reads to their cell of origin using the UMI tags, aligned these to the mm10 transcriptome (to which we added the tdTomato-WPRE sequence to detect tdTomato-expressing cells) using STAR (v.2.5.1b) and reported cell-specific gene expression count estimates. All subsequent analyses were performed in R v.3.6.0 using the Seurat (v3) package (Butler et al., 2018). Genes were considered to be ‘expressed’ if the estimated (log_10_) count was at least 0.1. Primary filtering was then performed by removing from consideration: cells expressing fewer than 50 genes and cells for which mitochondrial genes made up greater than 10% of all expressed genes. PCA decomposition was performed and, after consideration of the eigenvalue ‘elbow-plots’, the first 20 components were used to construct the UMAP plot, showing 6 distinct clusters. Cluster specific gene markers were identified using a wilcoxon rank sum test and the top 10 or 20 genes ranked by logFC per cluster were used to generate a heatmap.

Samples CD133+ DNGR-1 traced and CD133+ non-traced were sequenced and analysed as detailed above. Samples were integrated using Seurat’s ‘IntegrateData’ function, after identifying 2000 anchor features (which did not include the tdTomato-WPRE sequence, excluding that its expression influenced UMAP construction and cellular clustering). Three clusters were designated using the expression of various markers. Cells belonging to cluster 2 were subsetted and reclustered, resulting in seven clusters. Clusters 4 and 6 were removed due to the absence of ependymal cell markers (135 cells in total, of which 7 were CD133+ DNGR-1 traced, corresponding to 1.8% of all cells in cluster 2). Cluster markers was identified using a Wilcoxon Rank Sum test and used the package ‘ClusterProfiler’ (Wu et al., 2021) used to identify enriched Gene Ontology biological processes per cluster.

### Quantification and statistical analysis

All statistical analyses were performed using GraphPad Prism software (GraphPad). Normal distribution within each group was first confirmed using the Kolmogorov-Smirnov test. Statistical significance between two groups was determined using an unpaired two-tailed Student’s t test. Significance was assumed with *^∗^p<0.05*, *^∗∗∗∗^p<0.0001*.

## Data Availability

• This paper analyses existing, publicly available data. These accession numbers for the datasets are listed in the key resources table. • Bulk and single cell RNAseq datasets have been deposited at GEO and are publicly available as of the date of publication. Accession numbers are listed in the key resources table. • Any additional information required to reanalyse the data reported in this paper is available from the lead contact upon request.

## Supplemental information

Document S1. Figures S1–S7

Document S2. Article plus supplemental information
