# Supplementary material for: DNGR-1-tracing marks an ependymal cell subset with damage-responsive neural stem cell potential
Source: Dev Cell. 2022 Aug 22;57(16):1957–1975.e9. doi: 10.1016/j.devcel.2022.07.012 (PMC9616800; doi:10.1016/j.devcel.2022.07.012)
Supplement: Document S1. Figures S1–S7 [file mmc1.pdf]

Developmental Cell, Volume *57*

Supplemental information

DNGR-1-tracing marks an ependymal cell subset

with damage-responsive neural stem cell potential

Bruno Frederico, Isaura Martins, Diana Chapela, Francesca Gasparrini, Probir Chakravarty, Tobias Ackels, Cécile Piot, Bruna Almeida, Joana Carvalho, Alessandro Ciccarelli, Christopher J. Peddie, Neil Rogers, James Briscoe, François Guillemot, Andreas T. Schaefer, Leonor Saúde, and Caetano Reis e Sousa

Figure S1

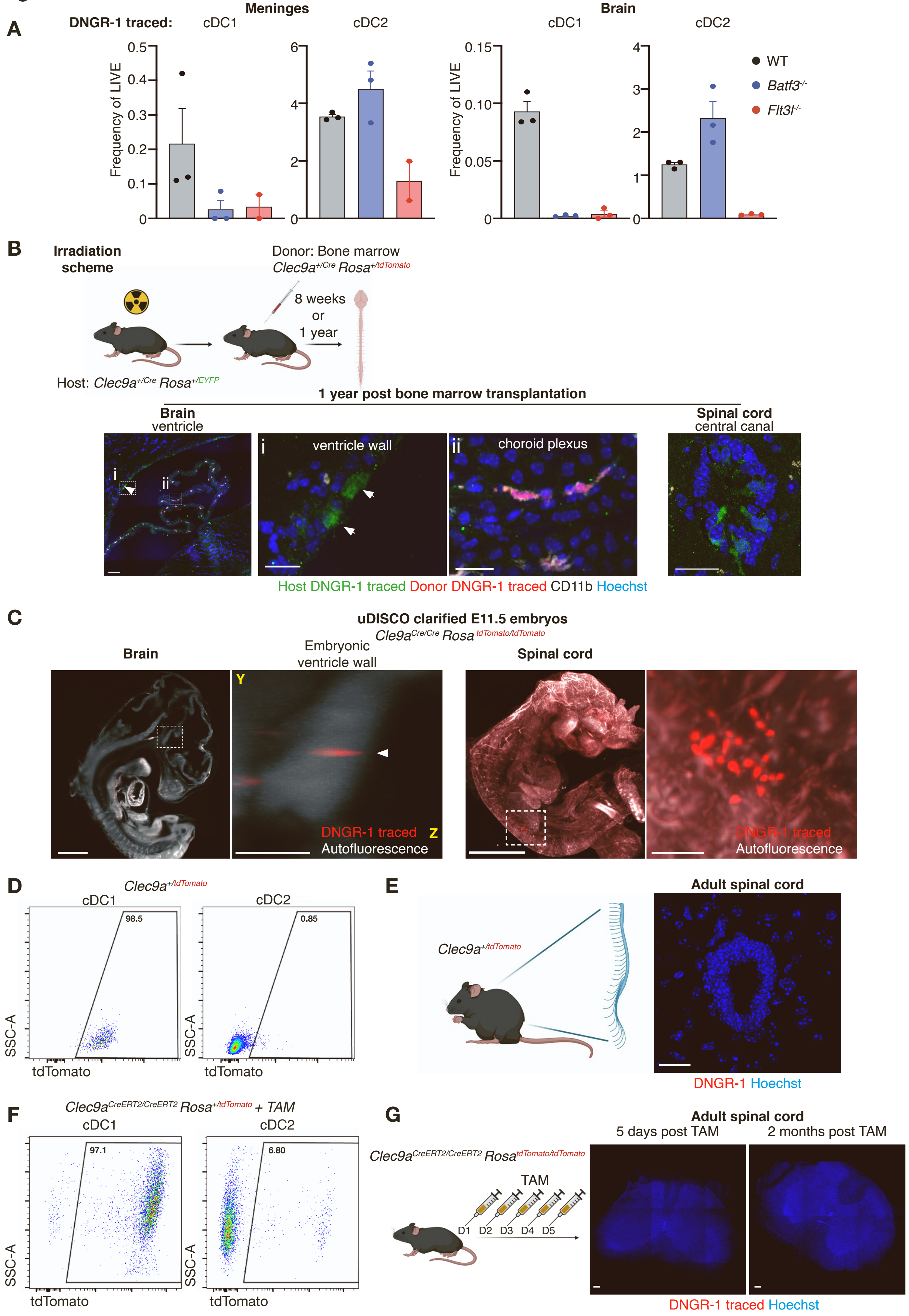

Clec9a+/Cre Rosa+/tdTomato
A
DNGR-1 traced:
Meninges
cDC1
cDC2
Brain
cDC1
cDC2
Frequency of LIVE
WT
Batf3-/-
Flt3l-/-
B
Irradiation scheme
Donor: Bone marrow
Clec9a+/Cre Rosa+/tdTomato
8 weeks or 1 year
Host: Clec9a+/Cre Rosa+/EYFP
1 year post bone marrow transplantation
Brain ventricle
i ventricle wall
ii choroid plexus
Spinal cord central canal
Host DNGR-1 traced Donor DNGR-1 traced CD11b Hoechst
C
uDISCO clarified E11.5 embryos
Cle9aCre/Cre Rosa tdTomato/tdTomato
Brain
Embryonic ventricle wall
Spinal cord
DNGR-1 traced
Autofluorescence
D
Clec9a+/tdTomato
cDC1
cDC2
98.5
0.85
SSC-A
tdTomato
E
Adult spinal cord
Clec9a+/tdTomato
DNGR-1 Hoechst
F
Clec9aCreERT2/CreERT2 Rosa+/tdTomato + TAM
cDC1
cDC2
97.1
6.80
SSC-A
tdTomato
G
Adult spinal cord
5 days post TAM
2 months post TAM
Clec9aCreERT2/CreERT2 RosatdTomato/tdTomato
TAM
D1 D2 D3 D4 D5
DNGR-1 traced Hoechst

Figure S2

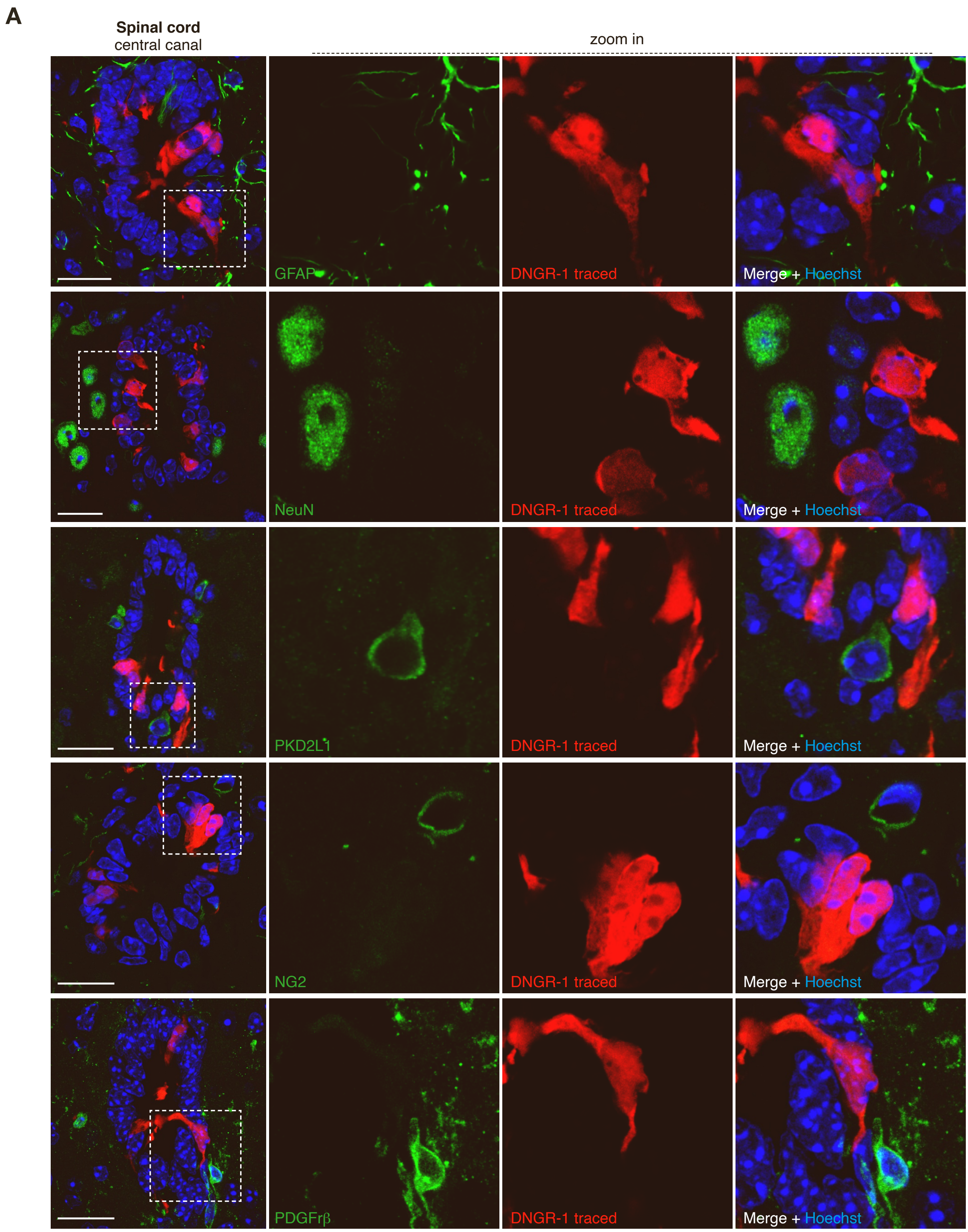
A
Spinal cord
central canal
zoom in
GFAP
DNGR-1 traced
Merge + Hoechst
NeuN
DNGR-1 traced
Merge + Hoechst
PKD2L1
DNGR-1 traced
Merge + Hoechst
NG2
DNGR-1 traced
Merge + Hoechst
PDGFrβ
DNGR-1 traced
Merge + Hoechst

Figure S3

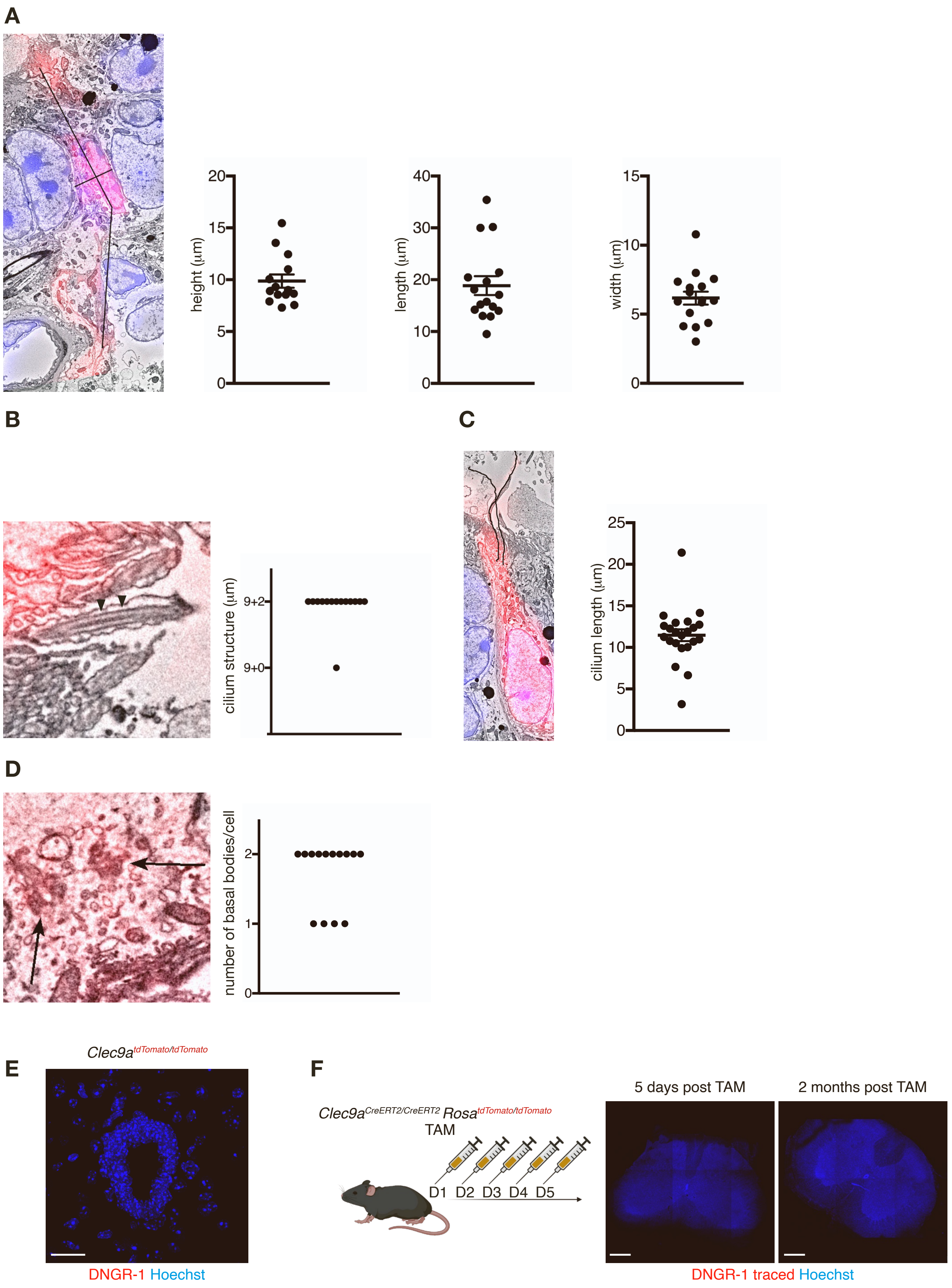
A
height (µm)
0
5
10
15
20
length (µm)
0
10
20
30
40
width (µm)
0
5
10
15
B
cilium structure (µm)
9+2
9+0
C
cilium length (µm)
0
5
10
15
20
25
D
number of basal bodies/cell
0
1
2
E
Clec9a tdTomato/tdTomato
DNGR-1 Hoechst
F
Clec9a CreERT2/CreERT2 Rosa tdTomato/tdTomato
TAM
D1 D2 D3 D4 D5
5 days post TAM
2 months post TAM
DNGR-1 traced Hoechst

Figure S4

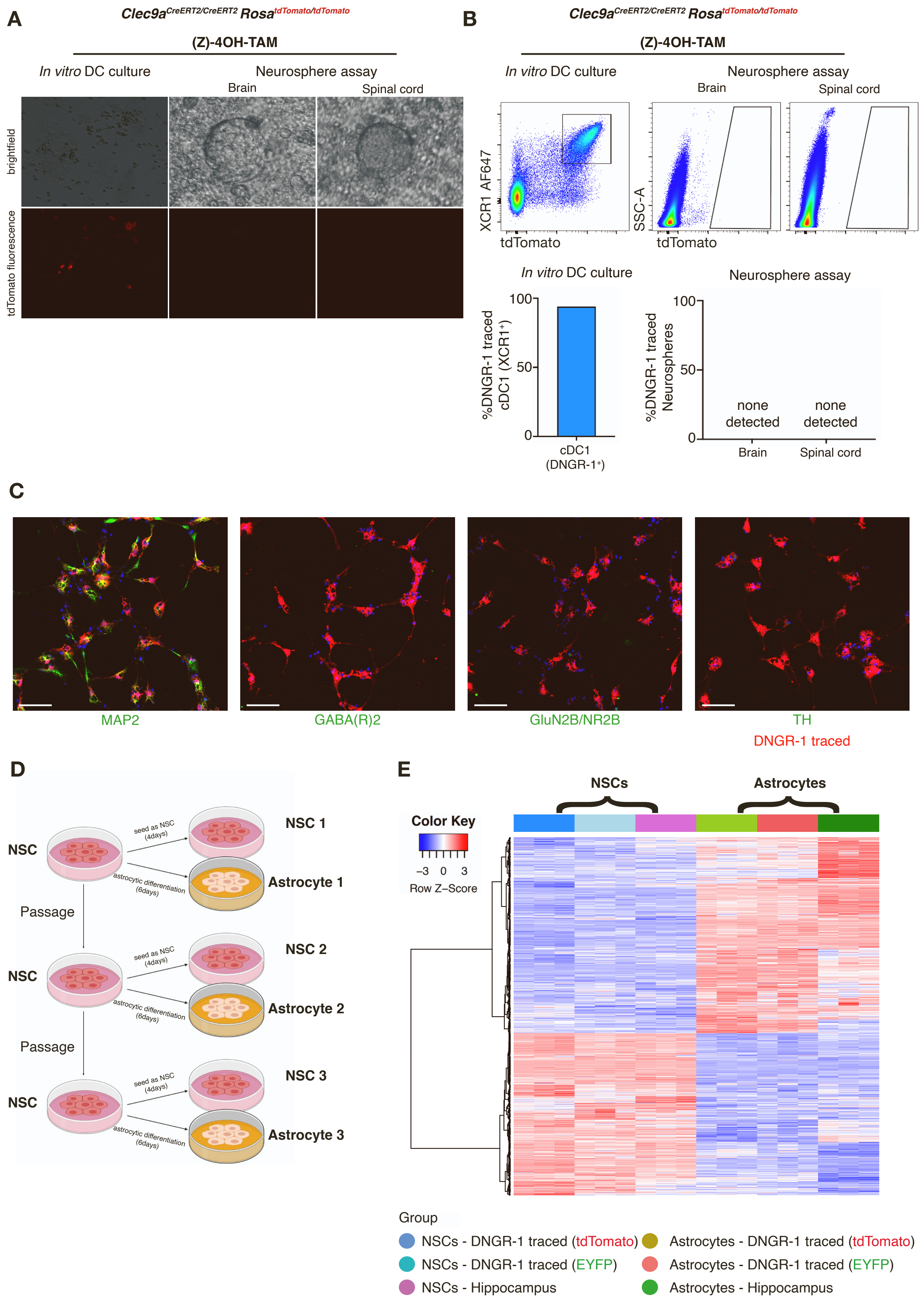

A
Clec9a CreERT2/CreERT2 Rosa tdTomato/tdTomato
(Z)-4OH-TAM
In vitro DC culture
Neurosphere assay
Brain
Spinal cord
brightfield
tdTomato fluorescence
B
Clec9a CreERT2/CreERT2 Rosa tdTomato/tdTomato
(Z)-4OH-TAM
In vitro DC culture
Neurosphere assay
Brain
Spinal cord
XCR1 AF647
tdTomato
SSC-A
tdTomato
In vitro DC culture
Neurosphere assay
%DNGR-1 traced cDC1 (XCR1+)
100
50
0
cDC1 (DNGR-1+)
%DNGR-1 traced Neurospheres
100
50
0
none detected
none detected
Brain
Spinal cord
C
MAP2
GABA(R)2
GluN2B/NR2B
TH
DNGR-1 traced
D
NSC
seed as NSC (4days)
NSC 1
astrocytic differentiation (6days)
Astrocyte 1
Passage
NSC
seed as NSC (4days)
NSC 2
astrocytic differentiation (6days)
Astrocyte 2
Passage
NSC
seed as NSC (4days)
NSC 3
astrocytic differentiation (6days)
Astrocyte 3
E
NSCs
Astrocytes
Color Key
−3 0 3
Row Z−Score
Group
NSCs - DNGR-1 traced (tdTomato)
NSCs - DNGR-1 traced (EYFP)
NSCs - Hippocampus
Astrocytes - DNGR-1 traced (tdTomato)
Astrocytes - DNGR-1 traced (EYFP)
Astrocytes - Hippocampus

Figure S5

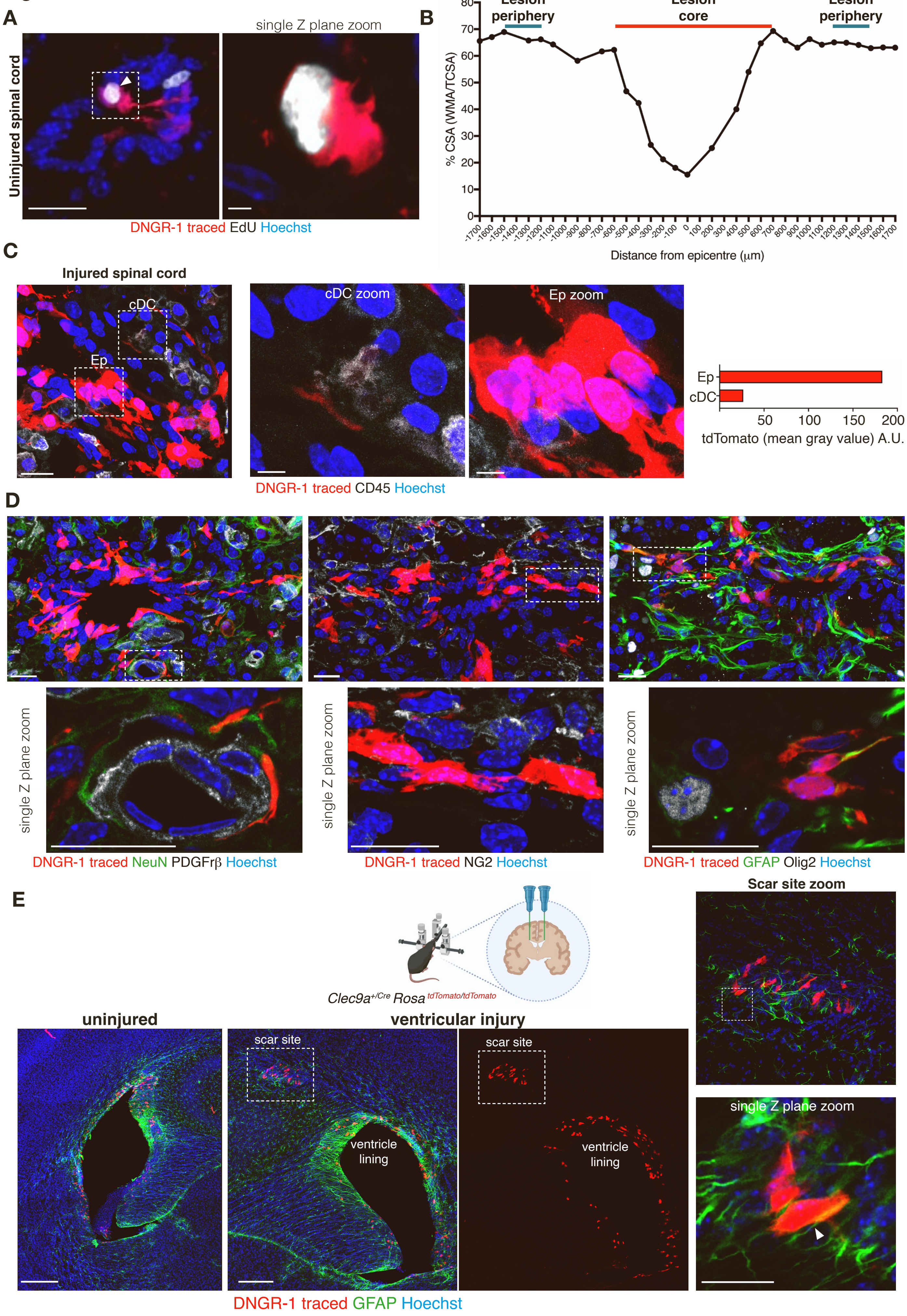

A
single Z plane zoom
Uninjured spinal cord
DNGR-1 traced EdU Hoechst
B
Lesion periphery
Lesion core
Lesion periphery
% CSA (WMA/TCSA)
0
10
20
30
40
50
60
70
80
-1700 -1600 -1500 -1400 -1300 -1200 -1100 -1000 -900 -800 -700 -600 -500 -400 -300 -200 -100 0 100 200 300 400 500 600 700 800 900 1000 1100 1200 1300 1400 1500 1600 1700
Distance from epicentre (μm)
C
Injured spinal cord
cDC
Ep
cDC zoom
Ep zoom
Ep
cDC
50 100 150 200
tdTomato (mean gray value) A.U.
DNGR-1 traced CD45 Hoechst
D
single Z plane zoom
DNGR-1 traced NeuN PDGFrβ Hoechst
single Z plane zoom
DNGR-1 traced NG2 Hoechst
single Z plane zoom
DNGR-1 traced GFAP Olig2 Hoechst
E
Clec9a+/Cre Rosa tdTomato/tdTomato
Scar site zoom
uninjured
ventricular injury
scar site
ventricle lining
scar site
ventricle lining
single Z plane zoom
DNGR-1 traced GFAP Hoechst

Figure S6

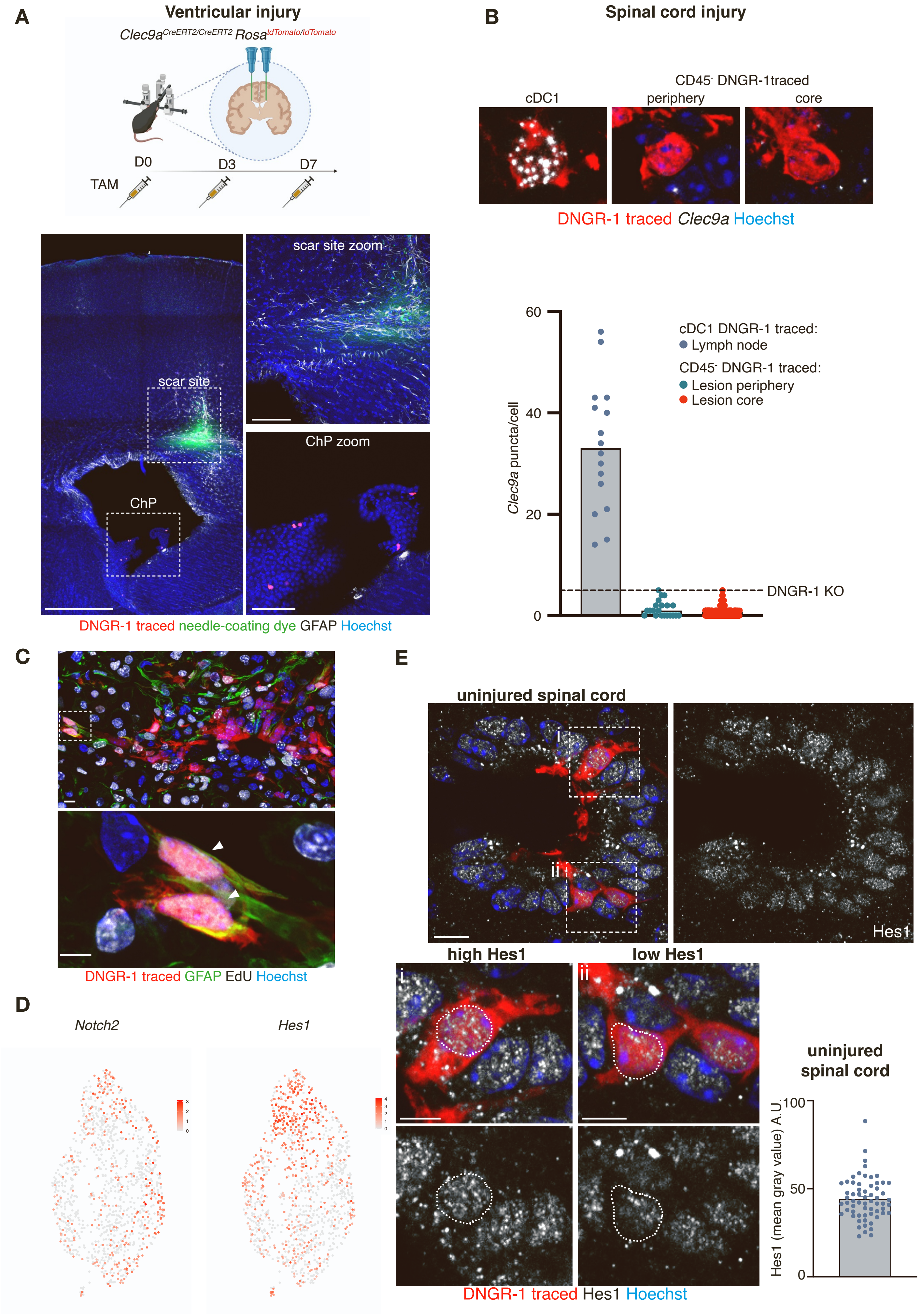

A
Ventricular injury
Clec9a^CreERT2/CreERT2 Rosa^tdTomato/tdTomato
D0
D3
D7
TAM
scar site zoom
scar site
ChP
ChP zoom
DNGR-1 traced needle-coating dye GFAP Hoechst
B
Spinal cord injury
CD45- DNGR-1traced
cDC1
periphery
core
DNGR-1 traced Clec9a Hoechst
60
40
20
0
Clec9a puncta/cell
cDC1 DNGR-1 traced:
Lymph node
CD45- DNGR-1 traced:
Lesion periphery
Lesion core
DNGR-1 KO
C
DNGR-1 traced GFAP EdU Hoechst
D
Notch2
Hes1
E
uninjured spinal cord
i
ii
Hes1
high Hes1
low Hes1
DNGR-1 traced Hes1 Hoechst
uninjured spinal cord
100
50
0
Hes1 (mean gray value) A.U.

Figure S7

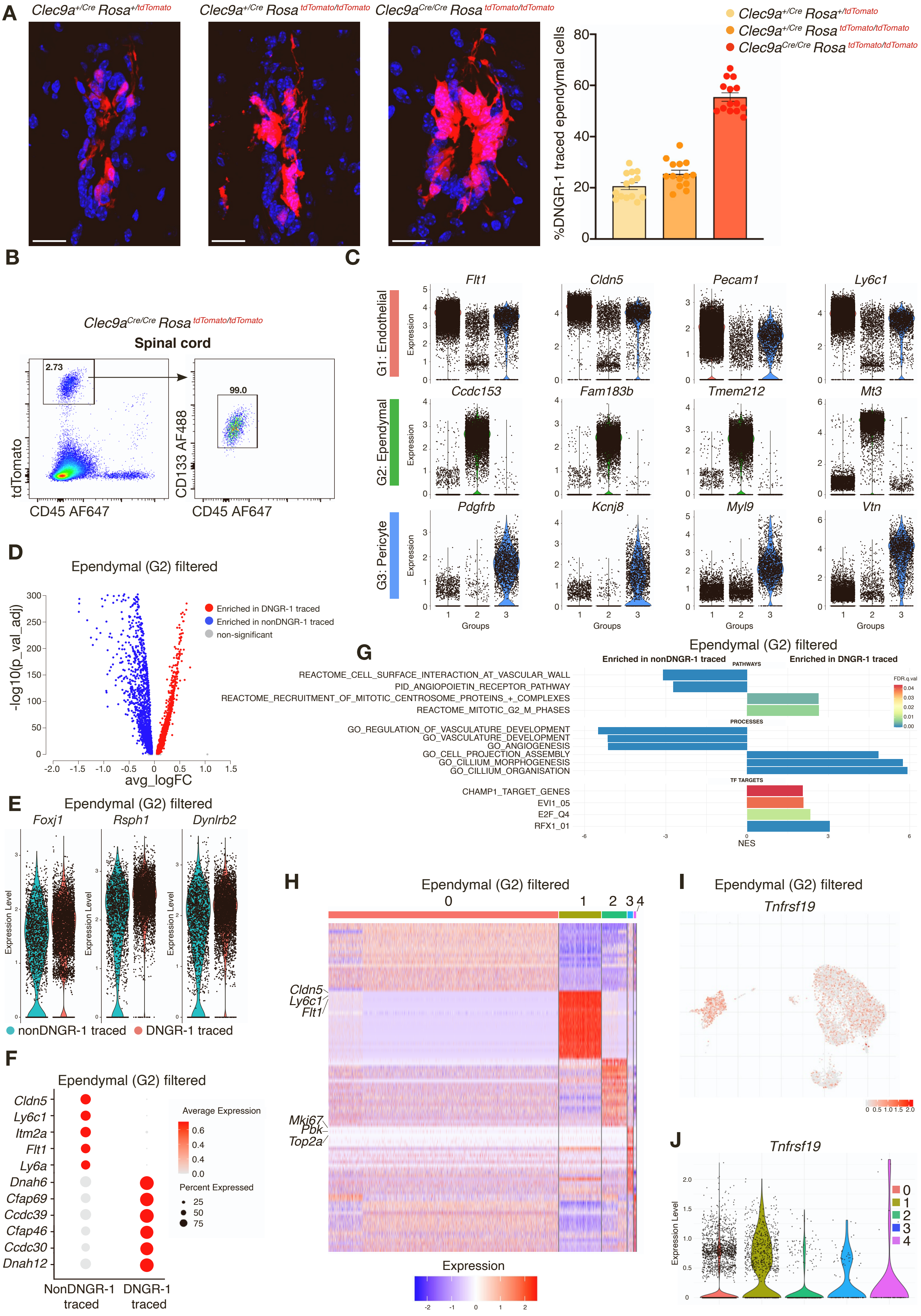
A
Clec9a+/Cre Rosa+/tdTomato
Clec9a+/Cre Rosa tdTomato/tdTomato
ClecCre/Cre Rosa tdTomato/tdTomato
%DNGR-1 traced ependymal cells
B
Clec9aCre/Cre Rosa tdTomato/tdTomato
Spinal cord
2.73
99.0
tdTomato
CD133 AF488
CD45 AF647
C
G1: Endothelial
G2: Ependymal
G3: Pericyte
Expression
Flt1
Cldn5
Pecam1
Ly6c1
Ccdc153
Fam183b
Tmem212
Mt3
Pdgfrb
Kcnj8
Myl9
Vtn
Groups
D
Ependymal (G2) filtered
-log10(p_val_adj)
avg_logFC
Enriched in DNGR-1 traced
Enriched in nonDNGR-1 traced
non-significant
E
Ependymal (G2) filtered
Foxj1
Rsph1
Dynlrb2
Expression Level
nonDNGR-1 traced
DNGR-1 traced
F
Ependymal (G2) filtered
Cldn5
Ly6c1
Itm2a
Flt1
Ly6a
Dnah6
Cfap69
Ccdc39
Cfap46
Ccdc30
Dnah12
Average Expression
Percent Expressed
NonDNGR-1 traced
DNGR-1 traced
G
Ependymal (G2) filtered
Enriched in nonDNGR-1 traced
Enriched in DNGR-1 traced
PATHWAYS
REACTOME_CELL_SURFACE_INTERACTION_AT_VASCULAR_WALL
PID_ANGIOPOIETIN_RECEPTOR_PATHWAY
REACTOME_RECRUITMENT_OF_MITOTIC_CENTROSOME_PROTEINS_+_COMPLEXES
REACTOME_MITOTIC_G2_M_PHASES
PROCESSES
GO_REGULATION_OF_VASCULATURE_DEVELOPMENT
GO_VASCULATURE_DEVELOPMENT
GO_ANGIOGENESIS
GO_CELL_PROJECTION_ASSEMBLY
GO_CILLIUM_MORPHOGENESIS
GO_CILLIUM_ORGANISATION
TF TARGETS
CHAMP1_TARGET_GENES
EVI1_05
E2F_Q4
RFX1_01
FDR.q.val
NES
H
Ependymal (G2) filtered
Cldn5
Ly6c1
Flt1
Mki67
Pbk
Top2a
Expression
I
Ependymal (G2) filtered
Tnfrsf19
J
Tnfrsf19
Expression Level

Supplemental figure legends

Figure S1 I CD45^{-} DNGR-1 traced are unrelated to cDCs. Related to Figure 2.

A. Flow cytometry quantification of DNGR-1 traced cDC1 or cDC2 in the meninges or choroid plexus of *Clec9a*^{Cre}*Rosa*^{LSLtdTomato} mice lacking *Batf3* or *Flt3l* (n=3 animals/group). Leucocytes were enriched in brain samples using a percoll gradient. Each dot represents one animal.

B. Irradiation scheme and immunofluorescence of CNS from irradiated *Clec9a*^{Cre}*Rosa*^{LSLEYFP} bone-marrow chimeric animals, 1 year post transplantation with *Clec9a*^{Cre}*Rosa*^{LSLtdTomato} bone marrow (n=3). Zoomed in areas show **i)** host-derived DNGR-1 traced ependymal cells (EYFP^{+}, green) lining the ventricle wall; and **ii)** a CD11b^{+} (white) donor-derived haematopoietic DNGR-1 traced cells (red) in the choroid plexus. Right, all DNGR-1 traced cells in the spinal cord central canal were of host origin (EYFP, green).

C. Optically clarified and light-sheet imaged E11.5 *Clec9a*^{Cre}*Rosa*^{LSLtdTomato} embryos showing DNGR-1 traced cells (red) embedded in the embryonic brain ventricle wall or spinal cord. Right, zoomed view of areas indicated with dashed outlines. Ventricle wall zoom is an orthogonal projection (YZ planes), spinal cord zoom is a maximum intensity projection. 10 embryos were analysed.

D. Flow cytometric analysis of spleens from *Clec9a*^{tdTomato} animals showing percentage of tdTomato-positive cDC1 and cDC2.

E. Immunofluorescence of the spinal cord central canal from an adult *Clec9a*^{tdTomato} animal. No DNGR-1 expressing cells (red) could be observed.

F. Flow cytometric analysis of spleens from *Clec9a*^{CreERT2}*Rosa*^{LSLtdTomato} animals given tamoxifen (TAM; 5 daily intraperitoneal injections) and examined 5 days after the last injection, showing percentage of DNGR-1 traced (tdTomato^{+}) cDC1 and cDC2.

G. Immunofluorescence of the spinal cord central canal from *Clec9a*^{CreERT2}*Rosa*^{LSLtdTomato} animals 5 days (mice from (F)) or 2 months post tamoxifen administration (5 daily doses; schematic on left). No DNGR-1 traced cells (red) were observed at either time point.

Scale bars, 1mm (**C**, brain), 500μm (**C**, spinal cord),100μm (**B, G,** zooms **C**) and 20μm (**E** and zooms **B**). (**D-E** are representative data of at least 3 animals analysed)

Figure S2 I DNGR-1 lineage-traced cells lining the central canal of the spinal cord are not astrocytes, oligodendrocyte precursors, neurons or pericytes. Related to Figure 3.

A. Immunofluorescence of spinal cord sections from *Clec9a*^{cre}*Rosa*^{LSLtdTomato} mice with GFAP, NG2, NeuN, PKD2L1, and PDGFrβ (green) and Hoechst (blue). No DNGR-1 traced ependymal cells (red) co-stained with any of the markers. Scale bars 20μm. Images are representative of at least 3 animals.

Figure S3 l Ultrastructural features of DNGR-1 traced spinal cord cells. Related to Figures 3 and 4.

Correlative light-electron microscopy from spinal cord sections from *Clec9a*^{Cre}*Rosa*^{LSLtdTomato}. DNGR-1 traced cells (red) were interrogated for several ultrastructure features, such as

A. dimensions;

B. cilium structure;

C. cilium length;

D. number of basal bodies per cell. Images are representative of at least 2 sections from 2 animals.

Figure S4 l Comparison of DNGR-1 traced, hippocampus-derived neural stem cells and their differentiated progeny in culture. Related to Ffigure 5.

A. Representative wide-field photographs of *in vitro* DC or neurospheres cultures grown from bone marrow or brain/spinal cord of *Clec9a*^{CreERT2}*Rosa*^{LSLtdTomato} animals, respectively, in the presence of 500nM (Z)-4OH-TAM. (Z)-4OH-TAM was replaced every two/three days. Whilst cDC cultures contained DNGR-1 traced cells, no DNGR-1 traced neurospheres were observed (>500 neurospheres/ organ analysed)).

B. Flow cytometric quantification of (**A**). Data are representative of two independent experiments.

C. Immunofluorescence of DNGR-1 traced neuronal differentiation cultures stained with antibodies against pan (MAP2) or mature neuronal markers TH (tyrosine hydroxylase), NR2B (NMDA receptor 2B) or GABA(B)R2 (GABA-B-receptor 2), all green; blue, DAPI; scale bar, 20 μm. Data are representative of two independent experiments.

D. Schematic representation of sample preparation for bulk RNA sequencing.

E. Heatmap of differential expressed genes between NSC and astrocyte cell fates.

Figure S5 l Differentiation of DNGR-1 traced ependymal cells after spinal cord injury is restricted to the astrocytic lineage. Related to Figure 7.

A. Immunofluorescence of central canal from uninjured *Clec9a*^{cre}*Rosa*^{LSLtdTomato} mice after EdU pulse. Arrowhead indicates a DNGR-1 lineage traced ependymal cell (red) which has incorporated EdU (white). Right, single optical Z plane of dashed boxed area showing nuclear localization of EdU signal.

B. White matter sparing at 7 days post spinal cord injury across the injury length, as described in the methods section. Each point equals one measurement. Values plotted derive from one

representative animal. CSA (Cross section area), WMA (white matter area), TCSA (total cross section area).

C. Immunofluorescence of injured spinal cord sections from *Clec9a*^{Cre}*Rosa*^{LSLtdTomato} mice with CD45 showing low tdTomato brightness in DNGR-1 traced cDCs and high tdTomato brightness in DNGR-1 traced ependymal cells. Scale bar, 20μm, zooms, 5μm.

D. Immunofluorescence of injured spinal cord sections from *Clec9a*^{Cre}*Rosa*^{LSLtdTomato} mice with NeuN or GFAP (both green), PDGFrβ, NG2 or Olig2 (all white) and Hoechst (blue). DNGR-1 traced ependymal cells (red) co-stained only with GFAP. Images are representative of at least 3 animals. Right, single optical Z planes of dashed boxed areas.

Scale bars 20μm.

E. Schematic representation of ventricular injury of *Clec9a*^{Cre}*Rosa*^{LSLtdTomato} mice and vibratome sections of uninjured or 28 days post-injury brains stained for GFAP (green) and with Hoechst (blue). Uninjured brains show showing DNGR-1 traced cells (red) residing exclusively in the ependymal layer and not expressing GFAP. Post-injury, DNGR-1-traced cells (red) can be seen dislodged from the ependymal layer and accumulating around the scar side where some cells express GFAP. Zooms show areas defined by dashed outlines. Arrowhead in lower panel zoom indicates a GFAP^{+} DNGR-1-traced cell in the scar site. Images are representative of three injured animals. Scale bars, 300μm (uninjured brain) 200μm (injured brain), 100μm (upper panel zoom) and 20μm (lower panel zoom).

Figure S6 I DNGR-1 traced ependymal cells do not re-express DNGR-1 after injury. Related to Figure 6.

A. Schematic representation of ventricular injury model applied to *Clec9a*^{CreERT2}*Rosa*^{LSLtdTomato} animals. Tamoxifen was injected intraperitoneally 1-2hrs prior to injury and at days 3 and 7 post injury. Vibratome section of a brain 28 days post-injury showing an astrocytic scar at the place where injury was inflicted (marked in this experiment by a needle-coating dye signal, green). No DNGR-1 traced cells were observed in the ventricular wall or at the scar site. DNGR-1 traced cells were only observed in the choroid plexus, consistent with their dendritic cell nature. Zooms showing areas defined by dashed outlines. Arrowhead indicates a GFAP^{+} DNGR-1-traced cell in the scar site. Images are representative of three injured animals. Scale bars, 500μm (overview) 100μm (zooms).

B. Detection of *Clec9a* transcripts by single molecule fluorescence *in situ* smFISH in lymph nodes or injured spinal cords from *Clec9a*$^{+/cre}$*Rosa*^{LSLtdTomato} animals. DNGR-1^{+} traced cDC1s (red) show abundant Clec9a expression (white puncta) as expected. Below, quantification of *Clec9a* puncta per cell indicated. Horizontal dotted line indicates background level as

determined by the maximum number of puncta observed in DNGR-1 deficient animals (*Clec9a*$^{cre/cre}$*Rosa*^{LSLtdTomato}) (n=5).

C. Immunofluorescence of DNGR-1 traced astrocytes in an injured spinal cord segment showing staining for EdU (arrowheads).

D. Feature plots showing projection of *Notch2* or *Hes1* expression by DNGR-1 traced ependymal cells on UMAP space. Low expression (grey) and high expression (red).

E. Immunofluorescence of spinal cord central canal from an uninjured *Clec9a*^{cre}*Rosa*^{LSLtdTomato} mouse and HES1 staining (white). Zooms of dashed boxed areas below show DNGR-1 traced cells (red) displaying **i)** high or **ii)** low HES1 levels. No HES1 negative DNGR-1 traced cells were observed. Right, quantification of Hes1 mean gray value intensity (a.u. arbitrary units). Each dot represents one cell. Quantification across at least 5 animals.

Scale bars, 10μm (**C**, **E**), 5μm (zooms **C**, **E**).

Figure S7 I Incomplete penetrance does not fully account for partial ependymal cell labelling and comparison between DNGR-1 traced and non-traced ependymal cells. Related to Figure 7.

A. Immunofluorescence images of spinal cord central canal from DNGR-1 tracer mice of different penetrance and quantification of ependymal cell labelling in each genotype. Red, DNGR-1 traced cells. Blue, Hoechst.

B. Flow cytometry enumeration of the proportion of spinal cord non-cDC-DNGR-1 traced cells ($CD45^-$, $tdtom^+$) stained with anti-CD133.

C. Expression of a selected set of transcripts from the integrated UMAP of $CD133^+$ DNGR-1 traced and $CD133^+$ non-traced cells (related to Figure 7D).

D. Volcano plot representing the differently expressed genes between DNGR-1 traced and non-traced ependymal cells (G2 filtered). Statistically significant genes enriched in DNGR-1 traced ependymal cells are coloured in red. Statistically significant genes enriched in nonDNGR-1 traced ependymal cells are coloured in blue. Non-statistically significant genes are coloured in grey. Statistical significance threshold = ($p<0.05$)

E. Violin plots showing expression of canonical ependymal cell genes in DNGR-1 traced and non-traced ependymal cell (G2 filtered populations).

F. Dot plot showing expression of selected top-differentially expressed genes between DNGR-1 traced and non-traced ependymal cells (G2 filtered).

G. GSEA analysis showing significant differential enrichment of selected Pathways, Processes and TF targets between DNGR-1 traced and non-traced ependymal cells (G2 filtered).

H. Heatmap showing the top 20 differentially expressed genes amongst the clusters belong to group 2 (related to Figure 7G).

I. Feature plot showing projection of *Tnfrsf19* transcripts on the UMAP space of ependymal (G2 filtered) population. Low expression (grey) and high expression (red).

J. Violin plot showing distribution of *Tnfrsf19 transcripts* across the different ependymal cell clusters (G2 filtered) population.
